# Oil‐Coated Nanoplastics Induce Rapid Membrane Disruption and Severe Intestinal Injury

**DOI:** 10.1002/advs.202520935

**Published:** 2026-03-18

**Authors:** Ruwen Xie, Gulimire Yilihan, Qiong Chen, Dachuan Lin, Zhen Liu, Mengyi Yuan, Yujia Wang, Haoteng Xu, Weishang Zhou, Wanxin Gong, Yueer Li, Chen Peng, Tong Yang, Peng Gao, Qing Liu, Xin‐Hua Feng, Mu Xiao, Chao Jiang

**Affiliations:** ^1^ MOE Key Laboratory of Biosystems Homeostasis & Protection and Zhejiang Key Laboratory of Molecular Cancer Biology Life Sciences Institute Zhejiang University Hangzhou China; ^2^ Department of Environmental Health Harvard T.H. Chan School of Public Health Harvard University Boston Massachusetts USA; ^3^ Department of Biological Sciences Clemson University Clemson South Carolina USA; ^4^ Center For Human Genetics Clemson University Greenwood South Carolina USA; ^5^ Cancer Center Zhejiang University Hangzhou Zhejiang China; ^6^ State Key Laboratory for Diagnosis and Treatment of Infectious Diseases First Affiliated Hospital Zhejiang University School of Medicine Hangzhou Zhejiang China

**Keywords:** exposure risk assessment, intestinal toxicity, Micro‐ and nanoplastics, oil‐plastic interactions

## Abstract

Plastic food containers used in food delivery may pose underrecognized health risks by releasing micro‐ and nanoplastics (MNPs), especially when in contact with oil‐rich foods. Here, we show that cooking oil dramatically increased MNP release from polypropylene (PP) and polyethylene (PE)‐coated containers within 3 min of microwave heating, reaching up to 1.52 × 10^14^ particles/L, approximately 125‐fold higher than with water. Concurrently, the co‐release of plastic additives and heavy metals increased by up to 309‐fold. Soybean oil‐derived PP nanoplastics were four times more cytotoxic (IC_50_ = 18.7 µg/mL) than water‐derived particles (IC_50_ = 77.1 µg/mL), likely due to oil‐film coatings, a positive surface charge (+7.37 mV), altered surface properties, and co‐extracted additives. Fluorescence and transmission electron microscopy revealed rapid membrane disruption within 5 min of exposure at 100 µg/mL. In vivo, oral exposure of mice to oil‐derived nanoplastics (50 mg/kg) for two weeks caused severe intestinal barrier damage and mucosal immune dysfunction. These adverse effects were partially alleviated by immune‐ and barrier‐targeted interventions. Benchmark dose modeling established safety thresholds of 0.4 µg/mL at the molecular level and 15.83 mg/kg for tissue injury. Alarmingly, modeled five‐year gastrointestinal burdens and reported human tissue MNP levels exceeded molecular safety thresholds by up to 1107‐fold, though not tissue injury thresholds. These findings highlight the urgent need for stricter regulations on oil‐contact plastics and the development of safer food packaging materials.

## Graphical abstract

Oil‐rich food contact dramatically amplifies MNP release from plastic takeout containers, producing oil‐coated nanoplastics with altered surface properties and rapid membrane‐disruptive effects. These particles cause severe intestinal barrier damage and immune dysfunction in mice, and risk modeling suggests that long‐term gastrointestinal burdens may exceed molecular safety thresholds.

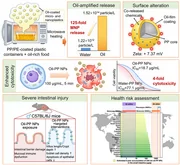

## 1 Introduction

Microplastics (MPs; < 5 mm) and Nanoplastics (NPs; < 1000 nm) have emerged as a pervasive yet poorly understood class of contaminants in the human food chain [1]. Their presence in commonly consumed products raises growing concerns about gastrointestinal exposure and associated health risks [2, 3, 4, 5, 6]. Among these plastic packaging materials, polypropylene (PP) and polyethylene (PE) are among the most widely used, owing to their durability, lightweight nature, and thermal resistance. However, the frequent use of plastic food‐contact materials in real‐life scenarios, such as heating, storage, and transportation, raises concerns about the release of micro‐ and nanoplastics (MNPs) and chemical additives, particularly when they interact with certain types of food [7, 8, 9]. Although substantial progress has been made in elucidating the toxicological mechanisms of MNPs, most studies rely on simplified experimental models, often using monodisperse polystyrene beads at concentrations far exceeding relevant exposure levels. While controlled laboratory conditions are valuable for uncovering mechanistic insights, they often fail to capture the complexity of real‐world exposure scenarios and the heterogeneous physicochemical properties of environmental MNPs. This mismatch limits our ability to accurately assess the health risks associated with MNP exposure under everyday conditions.

MNPs have been detected in a wide range of food products, including table salt [10], bottled water [11], seafood [12], edible oils [13], and packaged ready‐to‐eat meals [14]. Evidence increasingly demonstrates that MNPs can enter the human body through ingestion, with MNPs detected in the gastrointestinal tract contents and feces of both healthy individuals and patients [15, 16]. More importantly, smaller MNPs have been shown to directly cross biological barriers such as the gut epithelium, leading to systemic distribution [17]. Accumulation of MNPs has been confirmed in various human tissues and organs, including the placenta, liver, lung, and the bloodstream [18, 19, 20, 21]. Following this widespread exposure, MNPs, particularly NPs, pose significant health concerns due to their enhanced capacity to penetrate tissues and interact with cellular systems [22].

Experimental and epidemiological evidence indicate that MNPs can induce toxicity at multiple biological levels. In vitro studies have shown that MNPs trigger cellular stress responses [23], oxidative damage [24], and cytotoxicity [25]. In vivo animal models have linked ingestion of MNPs to metabolic disorders [26], DNA damage [27], inflammation [28], and disruption of intestinal barrier integrity [29]. Emerging human studies also suggest associations between elevated body burdens of MNPs and chronic conditions, including inflammatory bowel disease and metabolic syndromes [30]. However, a critical gap remains: most studies either investigate MNP toxicity under highly controlled experimental conditions or quantify MNP levels in human samples and infer disease associations, but few directly integrate experimentally derived safety thresholds with realistic exposure levels encountered in real‐world scenarios.

In everyday life, an important yet often overlooked source of MNPs is their release from plastic packaging in contact with oil‐rich foods. Such foods are increasingly prevalent in the global diet, especially in takeout meals such as stir‐fried dishes, fried foods, curries, and sauces, yet their contribution to MNP exposure remains understudied. In practice, MNPs interact with complex food matrices, especially fats and oils, which can alter their physicochemical properties, modulate bioavailability, and alter their toxicological behavior. Due to their non‐polar nature, cooking oils can markedly enhance the migration of MNPs, residual oligomers, and embedded additives from plastic food‐contact materials during heating, storage, or transportation [31, 32]. Despite clear evidence of these amplification mechanisms, most existing MNP studies still rely on water‐based simulants, largely neglecting oil‐based exposure scenarios, even though such conditions are explicitly recognized in regulatory testing frameworks [33].

In this study, we quantified and characterized MNPs released from PP and PE‐coated containers under three typical takeout scenarios, using deionized water as a control to explicitly assess the amplifying effects of cooking oil. We then evaluated the in vitro cytotoxicity of oil‐ and water‐derived MNPs and investigated their underlying mechanisms using relevant human cell models. Given their small size and high bioavailability, oil‐derived nanoplastics were selected as the most hazardous fraction for subsequent in vivo oral exposure experiments. We further elucidated the mechanisms of intestinal toxicity induced by oil‐derived nanoplastics and explored potential intervention strategies. By integrating the in vitro and in vivo results, we derived benchmark dose lower limits (BMDLs) for key molecular, cellular, and tissue‐level endpoints to define conservative safety thresholds. Finally, we estimated MNP exposure across 23 global regions and applied a one‐compartment input–output model to project five‐year small intestine MNP accumulation levels. These modeled burdens, together with reported MNP concentrations in human tissues, were comparable to or exceeded experimentally derived BMDLs. Collectively, this integrated framework directly links biological effects observed in cell and animal models with real‐world human MNP exposure levels.

## 2 Results

### 2.1 Physicochemical Properties of MNPs Released from Plastic Takeout Containers

**FIGURE 1:**
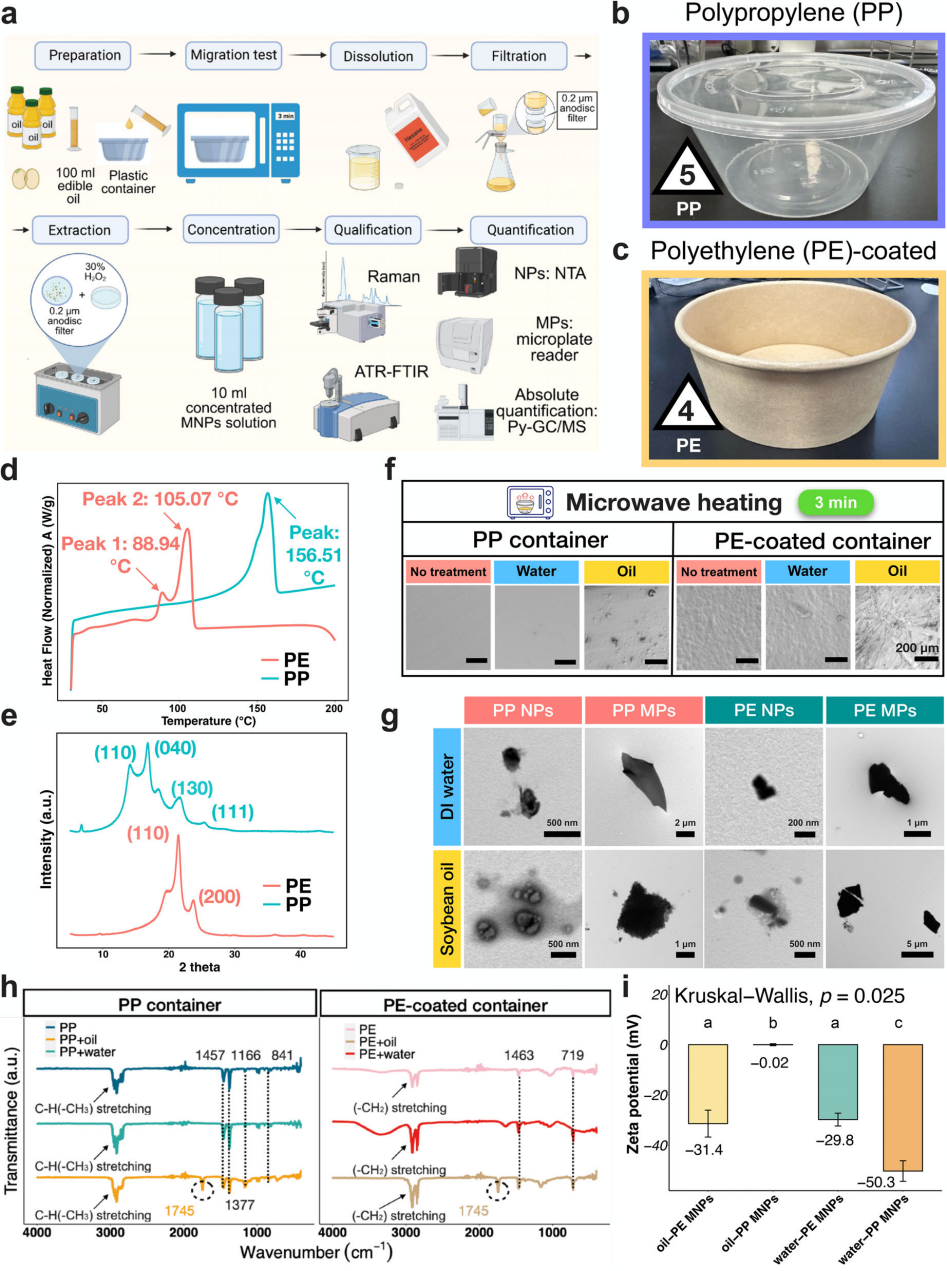
Extraction procedure and characterization of MNPs from plastic takeout containers. (a) Schematic of the extraction workflow for MNPs from cooking oil, along with the qualitative and quantitative analytical methods used. (b‐c) Representative images of two common food packaging types: (b) polypropylene (PP) and (c) polyethylene (PE)‐coated containers; (d) Differential scanning calorimetry thermograms of PP container and PE‐coated container; (e) X‐ray diffraction (XRD) patterns of PP container and PE‐coated container. (f) Scanning electron microscopy (SEM) images of the inner surfaces of PP and PE‐coated containers after exposure to deionized water or soybean oil and microwaving for 3 min. (g) Transmission electron microscopy (TEM) images of PP MPs, PP NPs, PE MPs, and PE NPs derived from PP and PE‐coated containers under soybean oil or DI water treatment. (h) Attenuated total reflectance Fourier‐transform infrared spectroscopy (ATR‐FTIR) profiles illustrate the plastics released from PP and PE‐coated containers exposed to soybean oil and DI water after microwave heating for 3 min. (i) Zeta potential of plastics released from PP and PE‐coated containers after 3 min of microwave heating at 800W. Measurements were performed in triplicate at 25°C in 10 mM NaCl. The *p*‐values for statistical differences were assessed using the Kruskal‐Wallis test with post hoc Games‐Howell tests. Different letters indicate significant differences (*p* < 0.05). Data are expressed as the mean ± standard deviation, n=3 per group.

We investigated the release of MNPs from two representative food packaging materials, polypropylene (PP) and polyethylene (PE) (Figure 1a), commonly used due to PP's thermal stability and PE's cost‐effectiveness (Figure 1b,c). Differential scanning calorimetry measurements showed notable differences in thermal stability between the PP and PE materials; the PP exhibited a significant melting peak at 156.51°C, indicating higher thermal stability (Figure 1d). X‐ray diffraction (XRD) analysis confirmed the crystalline structures, revealing distinct crystalline peaks (Figure 1e) [34].

We simulated three common scenarios: microwave heating (1, 3, and 5 min, Groups A‐C), transportation (15, 30, and 60 min, Groups D‐F), and leftover storage (1 and 5 h, Groups G‐H). We chose soybean oil as the main food simulant because of its widespread use, with deionized (DI) water serving as the control. Scanning Electron Microscopy (SEM) was employed to examine the treatment effects on the food‐contact surfaces of PP and PE‐coated containers. PP containers stayed smooth without treatment or with water microwaving, but oil microwaving caused damage and MNP release as early as 1 min, intensifying at 3 and 5 min (A–C; Figure 1f and Figure S1a). Oil also caused surface damage and triggered particle release during transport and leftover storage, while water had a minimal effect (D–H; Figure S1a). Similarly, the water treatment left the PE coating mostly intact, but oil‐filled containers showed substantial damage in all scenarios (Figure 1f and Figure S1b). For simplicity, MNPs released from PP containers filled with soybean oil or water were termed oil‐PP MNPs and water‐PP MNPs, respectively. Similarly, MNPs from PE‐coated containers were designated as oil‐PE MNPs and water‐PE MNPs.

Subsequently, we characterized the morphology of released MNPs from food containers. The released MNPs from PP and PE‐coated containers showed a diversity of particle sizes and shapes (Figure S2a,b). Transmission electron microscopy (TEM) (Figure 1g) and SEM were utilized for the detailed characterization of MPs and NPs (Figure S2c). Interestingly, oil‐PP NPs and oil‐PE NPs were coated with an oil film.

We analyzed the structural characteristics and functional groups of MNPs released from containers using Raman and Attenuated Total Reflectance Fourier Transform Infrared (ATR‐FTIR) spectroscopy. Raman analysis confirmed the identity of the released MNPs as PP and PE based on reference spectra (Figure S2d). Notably, ATR‐FTIR revealed an additional peak at 1745 cm^−^^1^ in oil‐PP and PE MNPs, indicating potential chemical modifications induced by microwave heating in the presence of oil (Figure 1h) [35, 36].

Zeta potential reflected the surface charge of plastic particles, related to their stability, aggregation, and interactions with other substances. After 3 min of microwave heating, significant differences in zeta potential were observed (*p*<0.05; Figure 1i). Water‐PP MNPs had a much lower zeta potential (‐50.3 ± 4.1 mV) than oil‐PP MNPs (‐0.025 ± 0.388 mV), indicating higher surface charge and potential instability in oil. In contrast, oil‐ and water‐PE MNPs had similar zeta potential. This shift toward a near‐neutral surface charge in oil‐PP MNPs is likely driven by an oil coating that replaces the surface hydration layer and exposes migrated additives, thereby altering the effective surface charge of the particles [13, 14].

### 2.2 Microwave Heating of Oil‐Filled Plastic Containers Increases the Release of MNPs and Harmful Additives While Reducing Particle Size

Quantifying the release and particle size distribution of MNPs is crucial for evaluating their potential environmental and health impacts. MPs were quantified using a BioTek Cytation 3 microplate reader, capable of detecting particles as small as 6 µm, with blank controls for accuracy [37] (Figure S2e,f). NPs were analyzed by Nanoparticle Tracking Analysis (NTA) with a NanoSight NS500 equipped with a 532 nm green laser, detecting particles from 10 nm to 1 µm.

**FIGURE 2:**
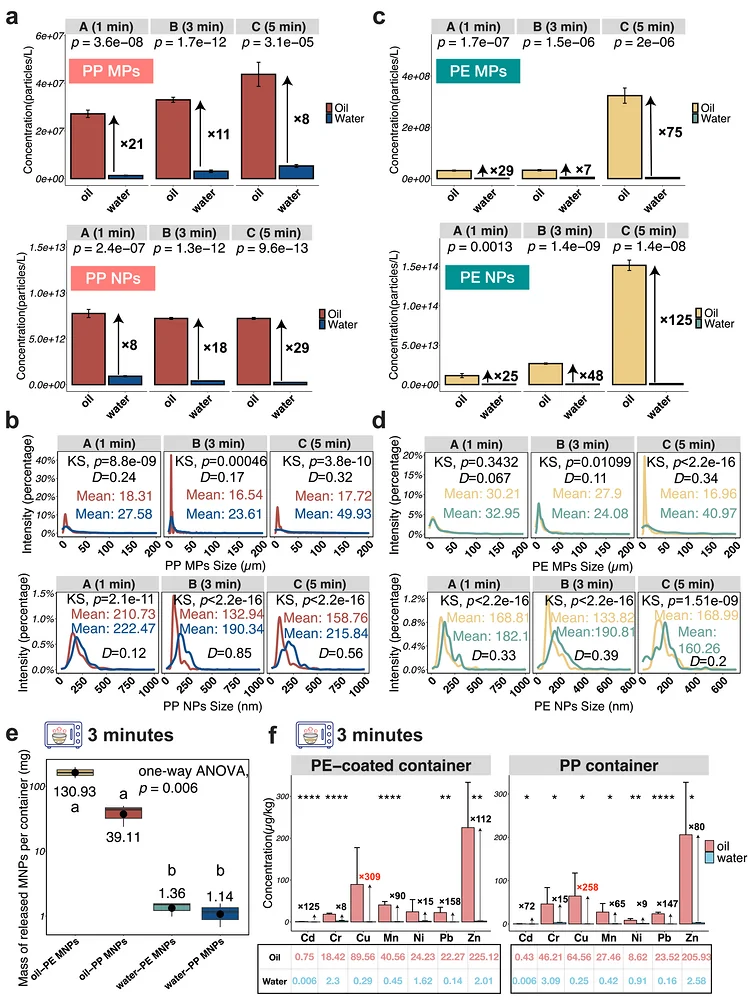
Abundance, size distribution, and mass of MNPs released, as well as heavy metal leaching, from PP and PE‐coated containers during microwave heating. (a) The abundance of PP MPs and PP NPs. (b) The size distribution of PP MPs and PP NPs. (c) The abundance of PE MPs and PE NPs. (d) The size distribution of PE MPs and PE NPs. Sample sizes: MPs (n = 10 per group) and NPs (n = 9 per group). (e) Total Mass of MNPs per container derived from 100 mL of oil and water after 3 min of microwave heating, n = 2 to 3 per group. (f) Heavy metal concentrations (µg/kg) released from PP and PE‐coated containers after 3 min of microwave heating (800W, 100 mL simulant; n = 3 per group). Metals were quantified by ICP‐MS with detection limits: Cd (0.008), Cr (0.006), Cu (0.04), Mn (0.01), Ni (0.3), Pb (0.01), Zn (0.3) µg/kg. The table below represents release in water and oil (µg/kg); fold‐changes above bars indicate the increase in oil relative to water. Statistical analysis used Student's t‐test (7 metals × 2 container types = 14 comparisons). Data are expressed as the mean ± standard deviation. The *p*‐values for statistical differences were assessed using the Student's t‐test, two‐sample Kolmogorov‐Smirnov test, and one‐way ANOVA test with Benjamini‐Hochberg correction for multiple comparisons, where applicable. *p < 0.05; **p < 0.01; ***p < 0.001; ***p < 0.001. Different letters indicate significant differences (*p* < 0.05).

In the microwaving group (1, 3, or 5 min at 800 W; oil temperature reaching 128°C and water 96°C), PP containers released significantly more and smaller oil‐PP MNPs than water‐PP MNPs (*p*<0.001; Figure 2a,b). After 5 min of heating, oil‐PP MPs reached 4.4 ± 1.0 × 10^7^ particles/L, 8 times higher than water‐PP MPs (*p* < 0.001). Oil‐PP NPs reached 7.2 ± 0.1 × 10^1^ ^2^ particles/L, 29 times higher than water‐PP NPs (*p* < 0.001), and had a smaller average size of 158.76 nm (*p* < 0.001). Notably, 85% of oil‐PP NPs were below 200 nm, compared with 57% for water‐PP NPs. PE‐coated containers had similar releasing dynamics (Figure 2c,d). Overall, under microwaving conditions, MNP release was at its highest in concentration and smallest in particle size, followed by simulated transportation and leftover food scenarios (Figure S3a–d; *p* < 0.01, Kruskal‐Wallis test and post‐hoc Games‐Howell tests). This enhanced release is driven by oil‐based microwave heating, which accelerates polymer softening and surface erosion (Figure 1f), reduces surface hardness, and increases lipophilicity, thereby promoting particle detachment.

Using Py‐GC/MS, we quantified the mass of MNPs released into 100 mL of soybean oil or water in PP and PE containers microwaved for 3 min. This duration was chosen to reflect typical household use while avoiding visible plastic deformation. To minimize interference from native fatty acids in the oil, we applied a pre‐treatment and analytical procedure optimized to reduce background lipid signals and reliably detect pyrolysis peaks specific to PE polymers [38] (Methods). In short, oil‐PE and PP MNPs showed the highest release (130.93 ± 68.29 mg and 39.11 ± 13.64 mg per container; *p* < 0.01; Figure 2e; Figure S3e). These results were consistent with particle counting data.

To test if the soybean oil‐induced hyper‐release of MNPs can be generalized, we microwave‐heated PP containers with four additional types of cooking oils (blend, palm, peanut, sunflower) for 3 min, all of which resulted in significantly higher MNP release compared to water (*p* < 0.0001; Figure S4a,b). Interestingly, soybean oil induced the highest release of PP MPs (3.26 ± 1.1 × 10^7^ particles/L) and PP NPs (7.87 ± 2.85 × 10^12^ particles/L), with the smallest average sizes (9.41 µm and 133.13 nm). Compared to other oils, soybean oil's higher unsaturated fatty acid content may promote oxidation during heating, accelerating PP degradation and increasing MNP release [39]. In contrast, peanut oil showed the lowest release. Oil‐PP MNPs were generally smaller than water‐derived counterparts (Figure S4a, b).

When released from plastic food containers, MNPs‐associated toxic additives, including heavy metals and pollutants, pose a significant concern [40]. ICP‐MS analysis showed minimal release of heavy metals into water but substantially higher levels in oil (Figure 2f). Oil‐filled PP containers released Zn at 205.9 µg/kg (80‐fold increase), Cu at 64.56 µg/kg (258‐fold increase), and Pb at 23.52 µg/kg (147‐fold increase). Oil‐filled PE‐coated containers had similar releasing dynamics, up to Cu at 89.6 µg/kg (309‐fold increase).

LC‐MS/MS analysis of methanol‐extracted samples of materials from PP and PE food containers, as well as the oil‐MNPs, identified a total of 3126 chemical features (confidence score ≥ 60; Figure S4c,d). Among the 40 most abundant chemicals, 33 were identified, including hazardous substances such as DEHP, avobenzone, and isoxadifen‐ethyl, commonly used as plasticizers and stabilizers (Tables S2 and S3; Figure S4e). Relative to the PP container extract, oil‐PP MNP extracts contained 667 additional detected features, indicating substantial changes in the extractable chemical profile following microwave heating in the presence of oil. This pattern is consistent with prior reports that oils can partition into hydrophobic polymer domains more effectively than water [15, 16], increasing additive mobility and promoting thermal degradation. Consequently, both MNPs and co‐migrating additives/metals are released at substantially higher levels under oil‐heating conditions.

### 2.3 Oil‐PP NPs Induce Cell Membrane Damage and Dose‐Dependent Cytotoxicity

**FIGURE 3:**
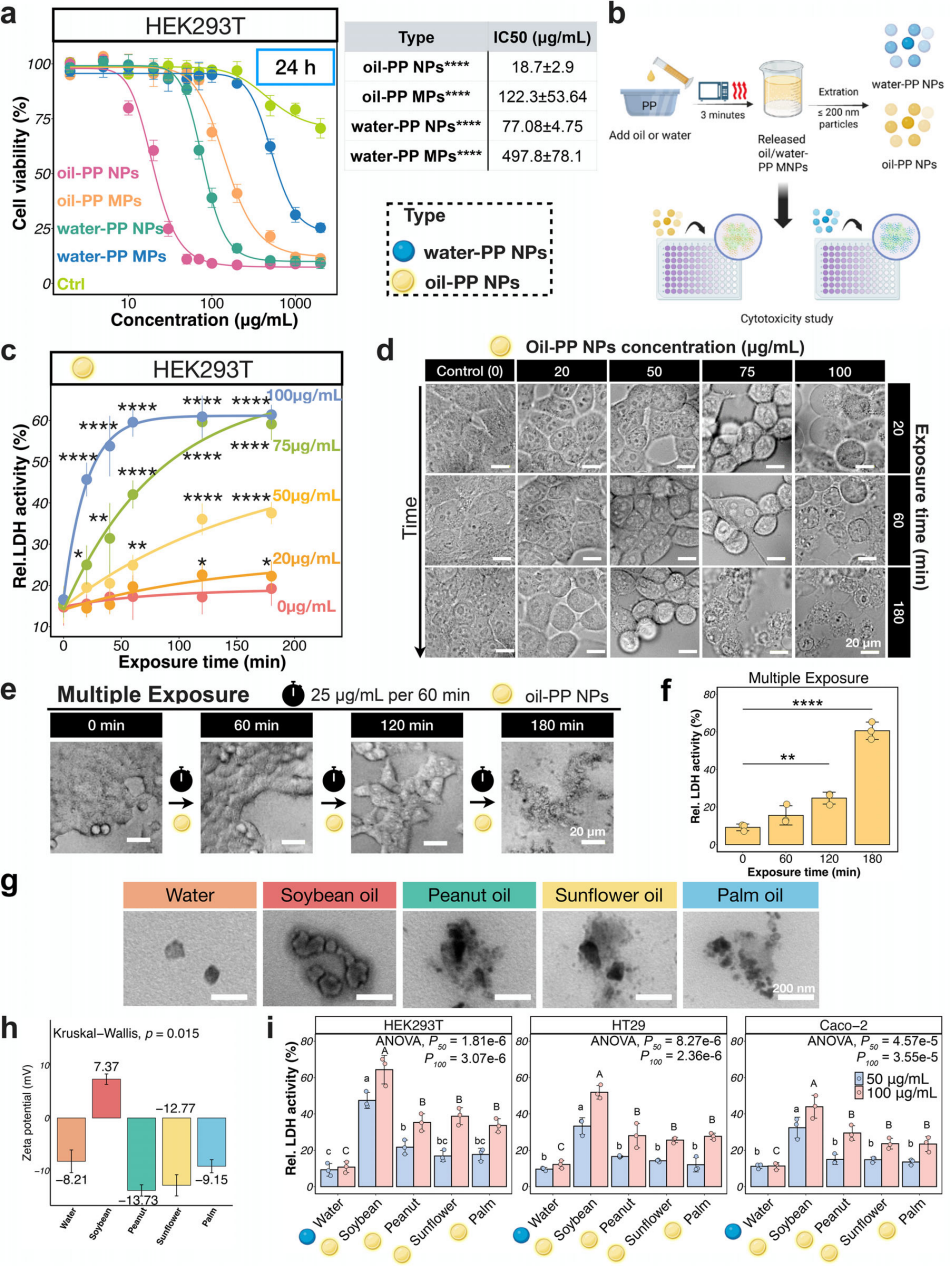
Cytotoxicity of different types of PP MNPs. (a) Cell viability of HEK293T cells after treatment with various MPs and NPs at concentrations ranging from 5 to 2000 µg/mL for 24 h. (b) Experimental design for PP NPs’ cytotoxicity in vitro. (c‐d) Short‐term exposure to high concentrations of oil‐PP NPs induced cell death. Panel c shows the statistical analysis of LDH release for each group. Representative phase‐contrast images are shown in panel d (scale bar: 20 µm). (e‐f) Repeated exposure to low concentrations of oil‐PP NPs also induced cell death, with representative phase‐contrast images shown (scale bar: 20 µm). Panel f provides the statistical analysis of LDH release. (g) TEM images of various types of PP NPs (scale bar: 200 nm). (h) Zeta potential of PP NPs in different oils. (i) Cytotoxicity of four types of oil‐PP NPs in HEK293T, HT29, and Caco‐2 cells after treatment with 50 and 100 µg/mL for 3 h. Blue circles denote water‐derived PP NPs, and yellow circles denote oil‐derived PP NPs.The *p*‐values for statistical differences were assessed using the Generalized Linear Model (GLM), Kruskal‐Wallis test, and **one‐way ANOVA with post hoc Tukey tests** (*p < 0.05; **p < 0.01; ***p < 0.001; ****p < 0.0001) where applicable. Groups that do not share a letter differ significantly (*p* < 0.05); lowercase denotes 50 µg/mL and uppercase denotes 100 µg/mL. Data are presented as the mean ± standard deviation, n = 3 per group.

Given the increased release and altered physicochemical properties of oil‐derived MNPs, we sought to evaluate their potential biological effects at the cellular level. Cytotoxicity assays were primarily conducted using HEK293T cells, a kidney‐derived line relevant to renal filtration and excretion of ingested MNPs. To assess sustained cytotoxicity, we used the CCK‐8 assay, which reflects long‐term metabolic activity and overall cell viability. After 24 h, oil‐PP NPs caused the most severe cytotoxicity compared to oil‐PP MPs, water‐PP NPs, and water‐PP MPs (*p* < 0.0001). The IC_50_ values were 18.7 ± 2.9, 122.3 ± 53.64, 77.1 ± 4.75, and 497.8 ± 78.1 µg/mL, respectively, confirming the elevated toxicity of NPs (Figure 3a).

To assess whether residual coating oil contributed substantially to cytotoxicity, we quantified the volume of oil film coating each particle (Methods; Table S4). When adding a 200 µL suspension of oil‐PP NPs at 100 µg/mL to cells, the maximum amount used in this study, the oil film accounted for only 0.136% of the total volume, or 0.271 µL, indicating that bulk oil exposure was negligible (Table S3).

Acute membrane damage was further evaluated using the lactate dehydrogenase (LDH) release assay. As NPs generally pose greater biological risks compared to MPs [43], subsequent in vitro experiments focused on oil‐PP NPs smaller than 200 nm (Methods; Figure 3b). Oil‐PP NPs induced clear dose‐ and time‐dependent cytotoxicity in HEK293T cells, as evidenced by elevated LDH activity (Figure 3c). At 100 µg/mL, LDH activity exceeded 60% within 120 min (*p* < 0.001), indicating significant membrane damage, whereas lower doses resulted in reduced toxicity (*p* < 0.05).

Consistent with these findings, cellular morphological analysis revealed distinct dose‐ and time‐dependent changes in cellular structure. Higher concentrations (75 and 100 µg/mL) caused rapid and severe membrane disruption, resulting in extensive cell detachment and death within 20‐60 min at 100 µg/mL (Figure 3d). These findings indicate that oil‐PP NPs rapidly compromise membrane integrity.

To better simulate real‐world consumption patterns involving repeated takeout food intake, cells were subjected to repeated low‐dose exposure to oil‐PP NPs (25 µg/mL added at 60‐min intervals). This exposure regimen results in a significant increase in cell death and LDH activity after the second exposure (*p* < 0.01), suggesting cumulative cytotoxic effects even at relatively low doses (Figure 3e,f).

To validate these observations in intestinal models, cytotoxicity assays were extended to gut‐derived HT29 and Caco‐2 cell lines, which represent the primary sites of MNP exposure. Nanoplastics were derived using five different cooking oils. TEM confirmed the presence of oil films on all oil‐PP NPs, while zeta potential analysis showed that soybean oil‐PP NPs carried a positive charge (+7.37±0.99 mV), in contrast to the negative charge observed for other oil‐derived particles (Figure 3g,h). Water‐PP NPs had the smallest mean size after filtration, whereas oil films appeared to promote aggregation of oil‐PP NPs (Methods; Figure S5a,b). At 50 and 100 µg/mL, oil‐PP NPs significantly increased LDH activity compared to water‐PP NPs in all cell lines (Figure 3i). Interestingly, soybean oil‐PP NPs consistently showed the highest cytotoxicity, potentially attributable to their positive surface charge.

Taken together, these results demonstrate that oil‐PP NPs exert both acute and cumulative cytotoxic effects across multiple cell and oil types, possibly driven by their altered surface charge, membrane‐disruptive properties, and enhanced cellular interactions.

### 2.4 Rapid Cellular Uptake and Membrane Disruption by Oil‐PP NPs Drive Acute and Sustained Transcriptomic Alterations

**FIGURE 4:**
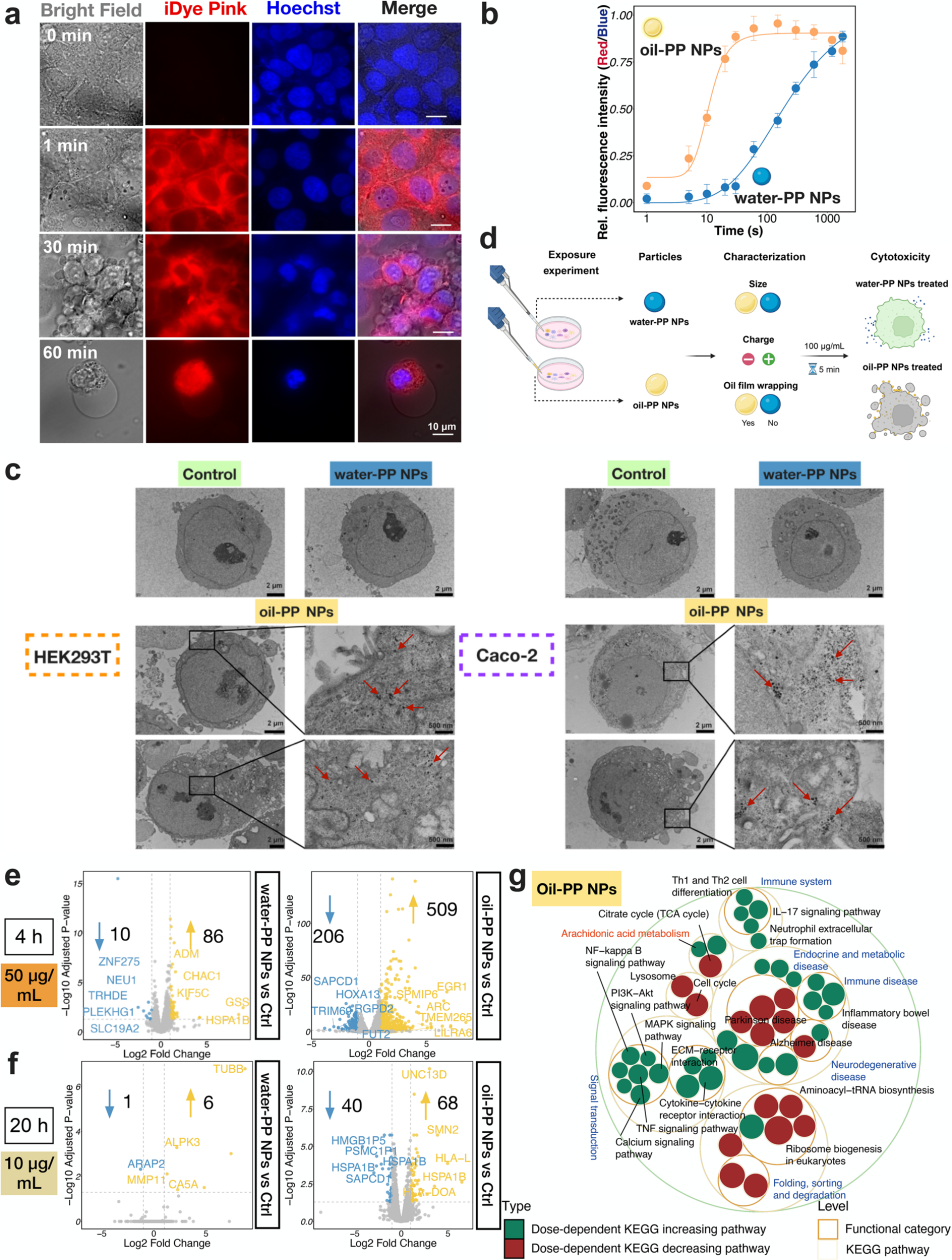
Fast cellular uptake and distinct transcriptomic responses of oil‐PP NPs versus water‐PP NPs (a) Oil‐PP NPs demonstrate rapid cell uptake when exposed to HEK293T cells. HEK293T cells were treated with 100 µg/mL oil‐PP NPs, and representative phase contrast and fluorescent images are shown. The scattering light signal of oil‐PP NPs was labeled with iDye Poly pink (red), and the nucleus was stained with Hoechst 33342 (blue). Scale bars represent 10 µm. (b) Relative fluorescence intensity (RFI) of FL‐oil/ water‐PP NPs (red) treated 293T cells, with nuclei (blue). (c) TEM images of cellular internalization of soybean oil‐PP NPs (100 µg/mL) in HEK293T cells (left) and Caco‐2 cells (right). Cells were fixed after treatment with 100 µg/mL oil‐PP NPs or water‐PP NPs for 5 min. Significant oil‐PP NPs were observed in the cell membrane and cytoplasm. Scale bars represent 2 µm for the original images and 500 nm for the zoomed‐in images. (d) Schematic diagram illustrating the rapid cell death triggered by oil‐PP NPs. (e,f) Volcano plots showing differentially expressed genes for water‐PP and oil‐PP NPs (10 and 50 µg/mL) versus control after 4 h (e) and 20 h (f) of exposure. BH‐corrected *p*‐adjusted <0.05 and |log_2_(FC)| ≥ 1. Genes with the top 5 |log_2_(FC)| values, either up‐regulated or down‐regulated, are labeled. (g) Gene‐function integration map tree related to differentially enriched pathways with significant (|ReporterScore| > 2, *p*.adj < 0.05) increases (green) or decreases (red) after 4 h of exposure to 50 and 100 µg/mL oil‐PP NPs. Data are expressed as the mean ± standard deviation, n = 3 per group.

We next investigated the real‐time cellular uptake behavior of oil‐derived polypropylene nanoplastics. To visualize nanoparticle internalization in HEK293T cells, we generated fluorescently labeled oil‐PP NPs (FL‐oil‐PP NPs) using polymer‐binding iDye Poly Pink (Figure S5c). NTA and dynamic light scattering (DLS) analysis showed that FL‐oil‐PP NPs had a consistent size distribution across various media, with a mean diameter of 177 nm and PDI values between 0.61 and 0.72 (Figure S5d–g). Laser confocal microscopy showed that at 100 µg/mL, FL‐oil‐PP NPs (red) adhered to the cell membrane within 1 min and quickly accumulated by 30–60 min, leading to cell swelling and membrane rupture, while nuclear (blue) structures remained mostly intact (Figure 4a). These findings suggest that oil‐PP NPs induced rapid cell death through fast cellular uptake and membrane disruption. To quantitatively compare uptake kinetics, time‐dependent relative fluorescence intensity (RFI) curves were generated for fluorescently labeled water‐derived PP NPs (FL‐water‐PP NPs) and FL‐oil‐PP NPs (Figure 4b). FL‐oil‐PP NPs showed markedly faster cellular uptake, reaching maximal intracellular fluorescence within 60 s.

TEM analysis further corroborated these findings. oil‐PP NP‐treated cells displayed ultrastructural damage within 5 min, including rupture, cellular swelling, and intracellular vesicle formation (Figure 4c). In contrast, control cells and water‐PP NP‐treated cells showed intact membranes and normal cytoplasmic morphology. These results indicate direct adherence of oil‐PP NPs to the cell surface at sites of membrane damage (Figure 4a,b), supporting a mechanism dominated by rapid particle‐membrane interactions rather than passive diffusion (Figure 4d).

To elucidate the downstream molecular consequences of oil‐PP NPs exposure, we conducted transcriptomic analysis, using water‐PP NPs as a control (Figure 4e–g; Figures S6 and S7). Exposure concentrations and durations were selected based on the IC_50_ value (18.7±2.9 µg/mL) for oil‐PP NPs. Lower doses (10 and 25 µg/mL for 20 h) were used to capture sustained transcriptional responses near the IC_50_, whereas higher doses (50 and 100 µg/mL for 4 h) were applied to probe cut gene expression changes.

Differential gene expression analysis revealed distinct dose‐ and time‐dependent transcriptomic responses. Under acute exposure conditions (50 µg/mL for 4 h), oil‐PP NPs induced substantially greater transcriptomic changes than water‐PP NPs, with 509 upregulated, 206 downregulated genes (Figure 4e). Enrichment analysis indicated significant disruption of lysosomal function and mitophagy‐related pathways, consistent with acute metabolic and mitochondrial stress (Figure S7a,b). In contrast, under prolonged low‐dose exposure (10 µg/mL for 20 h), both oil‐PP and water‐PP NPs triggered similar inflammatory and metabolic alterations, including activation of NF‐κB and Notch pathways and suppression of oxidative phosphorylation (Figure 4f; Figure S6c,d).

Specifically, at higher doses, oil‐PP NPs induced dose‐dependent genes that activated inflammatory pathways (e.g., MAPK, TNF, IL‐17), while suppressing metabolic processes, indicating acute mitochondrial dysfunction and cellular homeostasis disruption. Notably, enrichment of the arachidonic acid metabolism pathway suggested membrane damage driven by lipid peroxidation (Figure 4g; Figure S7a), consistent with the observed membrane‐disruptive effects of oil‐PP NPs [44]. In contrast, prolonged lower doses exposure upregulated glycine, serine, and threonine metabolism while continuing to suppress oxidative phosphorylation and ribosomal activity, indicating sustained mitochondrial stress and metabolic adaptation (Figure S6a; Figure S7b).

Notably, water‐PP NPs showed distinct dose‐dependent transcriptomic profiles under higher doses of exposure, characterized by activation of stress‐related pathways such as autophagy, apoptosis, and PI3K‐Akt signaling. Interestingly, the outcome of prolonged exposure at low concentrations is more similar to that of oil‐PP NPs (Figure S8a,b; Figure S9a–d).

Together, these results indicate that oil‐PP NPs caused acute mitochondrial and membrane disruptions at high doses, whereas both types of NPs contribute to chronic inflammatory damage and metabolic dysfunction under prolonged low‐dose exposure.

### 2.5 Simulated Daily Oral Exposure Disrupts the Intestinal Barrier and Immune Response in Mice

While in vitro assays provided mechanistic insights into the rapid membrane binding and disruption, and acute cytotoxicity of oil‐PP NPs. However, they could not fully recapitulate complex digestive, immune, and systemic responses in vivo. We, therefore, performed simulated gastrointestinal digestion and oral gavage experiments in C57BL/6J mice to evaluate NPs’ toxicity under more realistic exposure conditions.

The fate of the oil film of oil‐PP MNPs during gastrointestinal digestion remains an interesting question. We conducted in vitro oral–gastric–intestinal simulations and assessed their physicochemical properties. The zeta potential of oil‐PP NPs dropped from +7.37 ± 0.99 to −11.33 ± 0.93 mV after intestinal digestion (*p* < 0.05). A similar pattern was also observed for oil‐PP MPs (Figure S10a). These charge shifts were accompanied by reduced PDI (*p *< 0.05) and altered size distribution (Figure S10b,c), likely resulting from digestive biomolecule adsorption and improved colloidal stability. Despite these changes, ATR‐FTIR and TEM confirmed that the oil coating remained largely intact throughout digestion (Figure S10d,e), suggesting preserved structural integrity of oil‐film‐coated nanoplastic particles in vivo.

**FIGURE 5:**
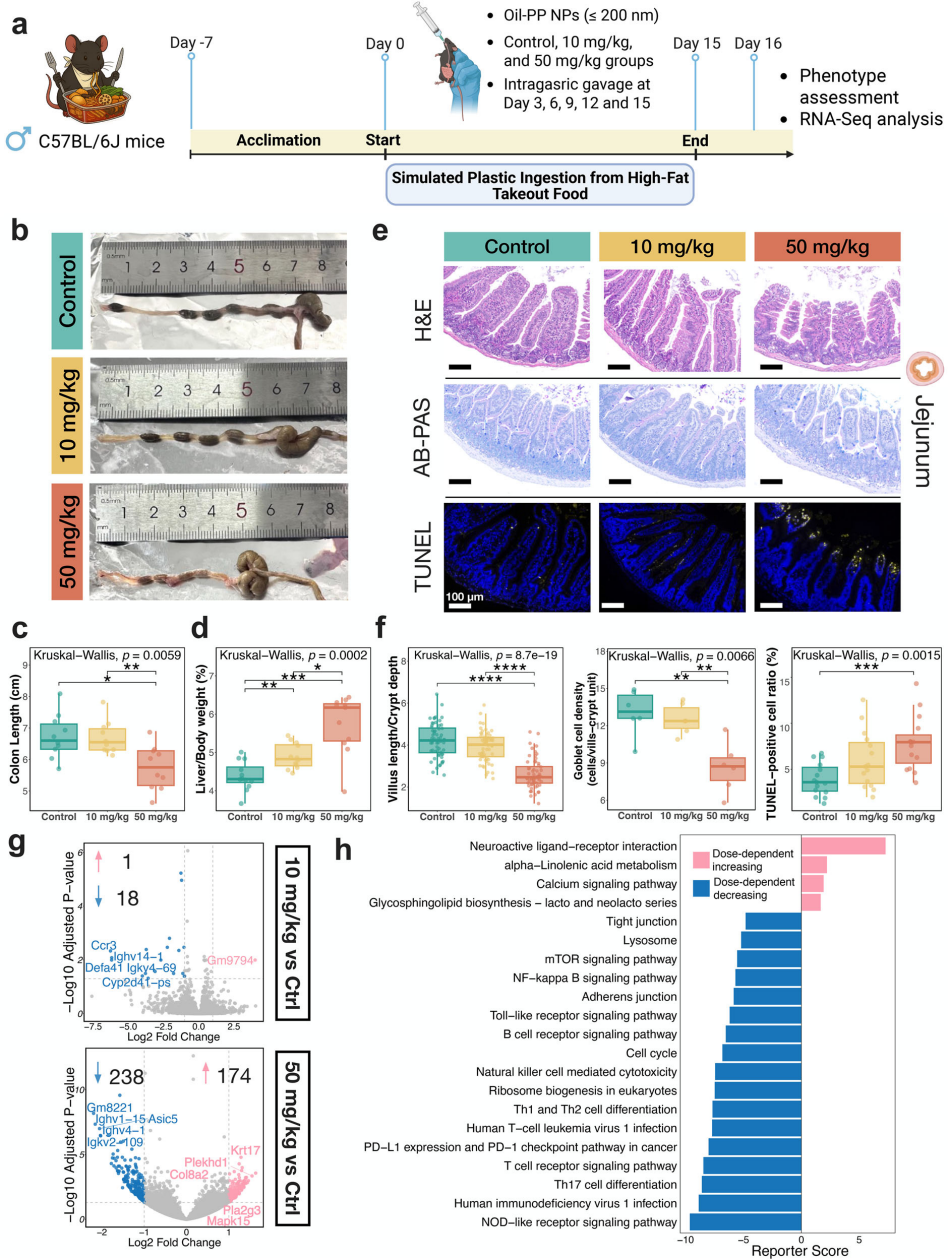
Ingestion of oil‐PP NPs induced intestinal injury and transcriptomic alterations in mice. (a) Experimental timeline: Male C57BL/6J mice were intragastrically gavaged with oil‐PP NPs (≤200 nm) at 0, 10, or 50 mg/kg on days 3, 6, 9, 12, and 15. Mice were sacrificed on day 16 for phenotypic and transcriptomic evaluation. **(b)** Representative images of colons from control, 10 mg/kg, and 50 mg/kg groups. **(c)** Boxplots illustrating dose‐dependent changes in colon length (n = 10 per group) and (d) liver‐to‐body weight ratio (n = 12 per group). **(e)** Histological analysis of jejunal sections using H&E staining (top; n=6), AB‐PAS staining for mucin‐producing goblet cells (middle; n=6), and TUNEL assay to detect apoptotic cells (bottom; n=5). Scale bar = 100 µm. **(f)** Quantification of goblet cell density, villus length‐to‐crypt depth ratio, and TUNEL‐positive cell ratio. **(g)** Volcano plots showing differentially expressed genes between the 10 mg/kg and control groups and between the 50 mg/kg and control groups. Genes significantly downregulated (blue) and upregulated (pink) are highlighted. BH‐corrected *p*‐adjusted <0.05 and |log_2_(FC)| ≥ 1. Genes with the top 5 |log_2_(FC)| values, either up‐regulated or down‐regulated, are labeled. (h) Bar plot showing significantly dose‐dependent enriched pathways (|Reporter Score| > 2, *p* < 0.05) in jejunal transcriptomes after 10 mg/kg and 50 mg/kg oil‐PP NPs exposure. Pathways with increased (pink) and decreased (blue) expression are indicated. The *p* values for statistical differences were assessed using the Kruskal‐Wallis test with post hoc Games‐Howell tests (*p < 0.05; **p < 0.01; ***p < 0.001; ****p < 0.0001).

To determine whether these structurally stable oil‐PP NPs exert adverse physiological effects, we performed a 2‐week exposure study in mice. Mice were gavaged every three days with 0, 10, or 50 mg/kg oil‐PP NPs, simulating exposure to particles released from around 0, 1, or 6 oil‐filled containers microwaved for 3 min (Figure 5a; Methods). Given the uncertainties in interspecies extrapolation and variability in real‐world intake, the doses used here should be regarded as high‐intake experimental scenarios rather than direct human equivalents. Mice exposed to 50 mg/kg exhibited significant colon shortening (*p*<0.01; Figure 5b,c) and weight loss (*p*<0.05; Figure S11a). Colon length showed a clear dose‐dependent reduction (*p*<0.01). Additionally, the liver‐to‐body weight ratio increased significantly at 50 mg/kg (*p* < 0.001; Figure 5d), indicating systemic stress or liver hypertrophy.

The jejunum was selected for histological and molecular analysis as it directly interfaces with ingested particles (Figure 5e–h). The high epithelial turnover and abundance of immune and mucus‐secreting cells in the jejunum make it particularly sensitive to barrier disruption. Oil‐PP NPs exposure resulted in clear, dose‐dependent intestinal barrier damage. Hematoxylin and Eosin (H&E) staining enabled visualization of general tissue morphology. The control jejunum displayed tall villi with intact brush borders and organized crypts. At 50 mg/kg, villus blunting, epithelial denudation, and crypt widening were prominent (Figure 5e). Quantitative analysis showed 7% and 38% reductions in the villus‐to‐crypt ratio at 10 and 50 mg/kg, respectively, compared to the control group (*p* < 0.001; Figure 5f). Alcian Blue‐Periodic Acid‐Schiff (AB‐PAS) staining, which targeted goblet cells and mucin production, revealed goblet cell depletion of 4% and 34% at the respective doses (*p* < 0.001). At the same time, TUNEL assays showed apoptosis at 113% in the 50 mg/kg group (*p* < 0.001), indicating a substantial compromise in barrier integrity.

At the molecular level, transcriptomic profiling of the jejunum showed predominant down‐regulation of immune‐ and barrier‐related genes in both the 10 and 50 mg/kg groups, with much broader gene suppression at 50 mg/kg (Figure 5g; Figure S11b). In line with the observed injury phenotypes, KEGG pathway enrichment analysis further demonstrated dose‐dependent (0‐50 mg/kg) suppression of immune signaling and barrier integrity pathways in the jejunum, including down‐regulated pathways related to mucosal immunity (e.g., NOD‐like receptor, Toll‐like receptor, T/B cell receptor signaling) and epithelial junctions, while only a few metabolic pathways were up‐regulated (Figure 5h; Figure S11c).

To further dissect the immune perturbations underlying these transcriptomic patterns, we systematically compared the dose‐dependent expression trends of annotated immune‐related genes in HEK293T cells (in vitro) and mouse jejunal tissue (in vivo) after exposure to oil‐PP NPs (Methods; Figure S11d). In vitro, immune‐related genes showed a bidirectional transcriptional response, with 86 genes upregulated and 160 downregulated. In contrast, in vivo responses were almost entirely immunosuppressive, with 306 immune‐related genes downregulated and only 2 upregulated.

### 2.6 Immune and Barrier‐Targeted Interventions Partially Mitigate Oil‐PP NP–Induced Intestinal Injury

Transcriptomic analysis revealed that oil‐PP NP exposure elicited the most pronounced perturbations in immune‐associated pathways (nod‐like receptor and T‐cell receptor signaling) and genes associated with tight junctions (Figure 5h). These findings prompted us to examine whether reinforcing immune sensing and epithelial barrier integrity could mitigate oil‐PP NP–induced intestinal injury.

**FIGURE 6:**
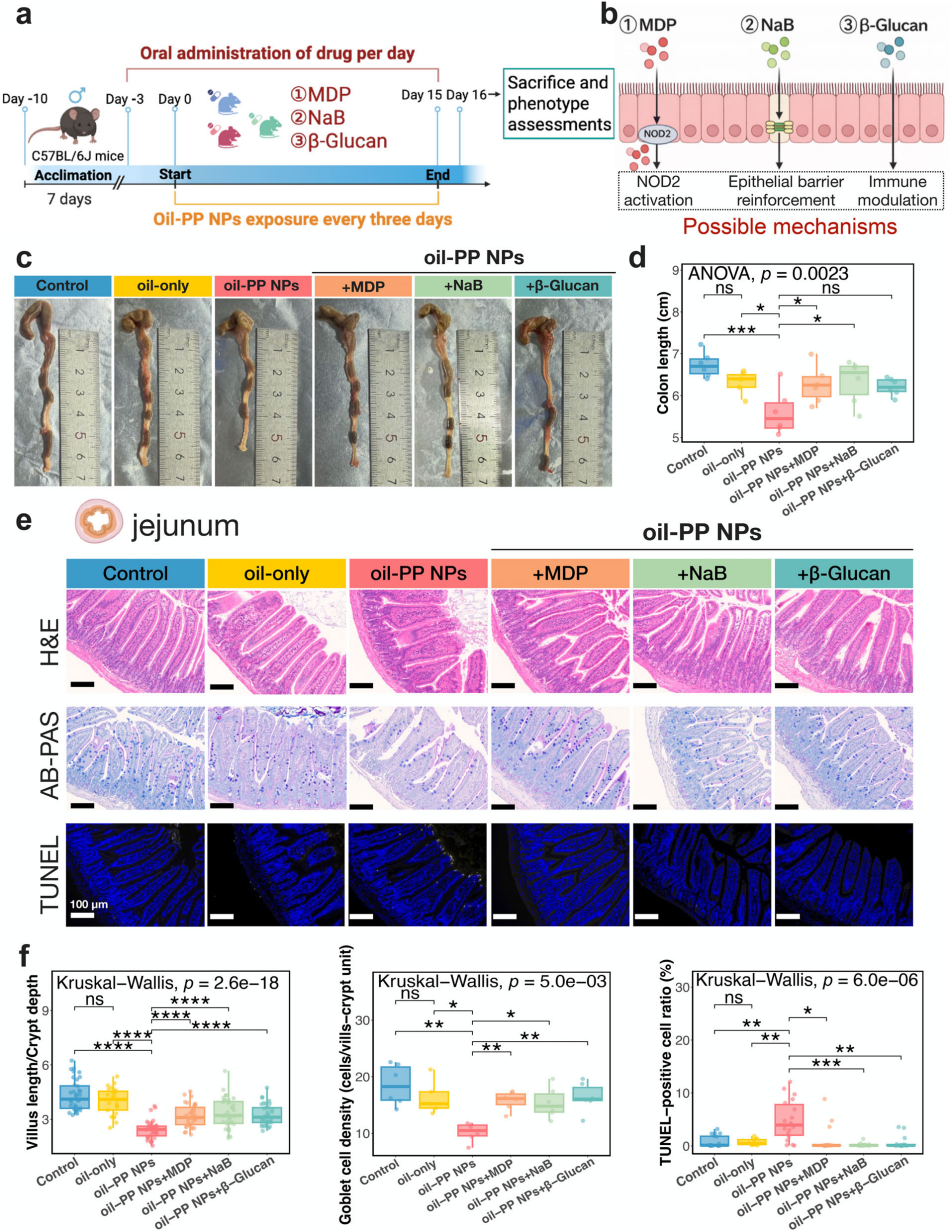
Targeted interventions partially mitigate the intestinal injuries in oil‐PP NP‐exposed mice. (a) Experimental timeline: Male C57BL/6J mice were exposed to oil‐PP NPs (25 mg/kg) every three days for two weeks. Daily muramyl dipeptide (MDP), sodium butyrate (NaB), or β‐glucan was co‐administered starting 3 days before the first NP exposure and continued throughout the exposure period. (b) Schematic of the intervention study design. (c) Representative colon images from the control, oil‐only, oil‐PP NP, and intervention groups (oil‐PP NPs + MDP, + NaB, + β‐glucan). (d) Quantification of colon length in each group. (e) Histological analysis of jejunal sections using H&E staining, AB‐PAS staining for mucin‐producing goblet cells, and TUNEL staining for apoptotic cells. Scale bar = 100 µm. (f) Quantification of villus length‐to‐crypt depth ratio, goblet cell density, and TUNEL‐positive cell ratio (oil‐only n = 5 per group; all other groups n = 6 per group). The *p* values for statistical differences were assessed using the one‐way ANOVA with post hoc Tukey tests and Kruskal‐Wallis test with post hoc Games‐Howell tests where applicable (*p < 0.05; **p < 0.01; ***p < 0.001; ****p < 0.0001).

Mice were orally exposed to oil‐PP NPs at 25 mg/kg every three days for two weeks. This dose was selected because it induced clear intestinal injury while avoiding the more severe immune suppression observed at 50 mg/kg. Muramyl dipeptide (MDP), sodium butyrate (NaB), or β‐glucan was administered beginning 3 days before the first NP exposure and continued throughout the exposure period (Figure 6a). MDP was used to target NOD2‐dependent immune functions [45, 46], NaB to reinforce epithelial barrier integrity [47, 48], and β‐glucan to modulate mucosal immune responses [49, 50] (Figure 6b).

As expected, oil‐PP NP exposure at 25 mg/kg significantly shortened colon length compared to the control (*p*<0.001; Figure 6c,d), whereas the oil‐only group did not show significant damage, indicating that the nanoparticles, in combination with the oil matrix, contributed to the injury. MDP and NaB treatments significantly alleviated colon shortening (*p*<0.05), suggesting at least partial restoration of intestinal integrity.

Histopathology corroborated these findings. Oil‐PP NP exposure induced marked epithelial injury, including villus blunting and crypt disorganization (*p*<0.0001; Figure 6e,f), which was significantly attenuated by all three interventions (*p*<0.0001). AB‐PAS staining showed goblet cell depletion and mucus barrier disruption in oil‐PP NP‐exposed mice (*p*<0.01), whereas each intervention significantly restored goblet cell density (*p*<0.05). Consistently, TUNEL assays confirmed that oil‐PP NP exposure increased apoptosis of intestinal epithelial cells (*p*<0.01), which was significantly reduced by MDP, NaB, and β‐glucan (*p*<0.05). The oil‐only group did not differ significantly from controls across these readouts, suggesting that oil alone did not drive detectable epithelial injury under these conditions.

Transcriptomic profiling indicated recovery following intervention. PCA showed clear separation between oil‐PP NP–exposed mice and controls (*q* < 0.05), whereas intervention groups shifted toward controls, most prominently with MDP (Figure S11e). Correspondingly, *Ripk2*, *Tab1*, and *Chuk* were significantly upregulated in the oil‐PP NPs + MDP group (*p* < 0.05; Figure S11f).

Collectively, these results demonstrate that immune‐ and barrier‐targeted interventions, including MDP, NaB, and β‐glucan, partially mitigate oil‐PP NP–induced intestinal injury, with restoration of innate immune signaling representing one potential molecular mechanism [51, 52].

### 2.7 Estimated and Observed Oil‐Derived MNPs in Humans May Exceed Safety Thresholds

To provide practical safety guidance, we established safety exposure thresholds for PP MNPs using benchmark dose (BMD) [53] analysis, as recommended by the USEPA and EFSA. We estimated benchmark dose lower limits (BMDLs) across molecular to phenotypic levels by integrating in vitro transcriptomic and cell viability data with in vivo transcriptomic and phenotypic outcomes.

**FIGURE 7:**
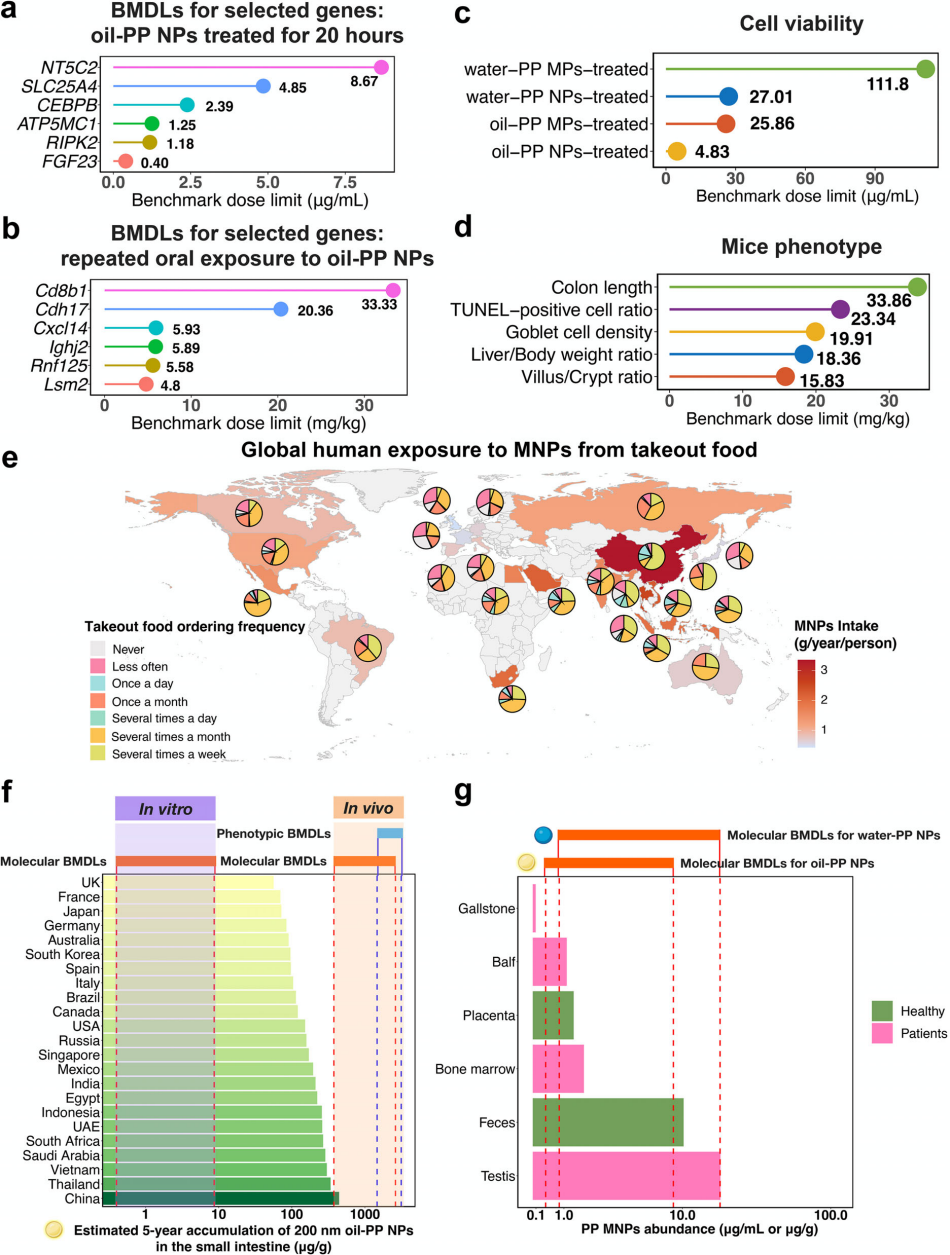
Estimated exposure safety thresholds for oil‐derived MNPs and their relevance to realistic human internal exposure levels. Lollipop chart of benchmark dose lower limits (BMDLs) for (a) selected dose‐responsive genes after 20 h treatment with oil‐PP NPs, (b) selected dose‐responsive genes in the mouse gavage study with oil‐PP NPs (see Figure S12c,e), (c) cell viability endpoints for different MNPs, and (d) in vivo phenotypic endpoints from the mouse gavage study. (e) Global MNP exposure (g/person/year) from PP containers across 23 regions. The full list of the 23 regions is provided in Table S6. (f) An estimated five‐year accumulation of 200 nm oil‐PP NPs in the small intestine across 23 regions under typical takeout‐food exposure scenarios, modeled using a single‐compartment input‐output approach, the same 23 regions in Figure 7e were used to ensure direct regional correspondence, and (g) Measured PP MNP concentrations in six human sample types from healthy donors and patients reported in previous studies, compared against molecular (red) and phenotypic (blue) BMDLs for oil‐ and water‐derived PP MPs and NPs.

At the molecular level, 214 and 596 dose‐responsive genes were identified for oil‐ and water‐PP NPs in vitro, and 662 for oil‐PP NPs in vivo (Tables S9–S11; Figure S12a–f). These genes were subjected to BMD analysis. In vitro, the lowest BMDL for oil‐PP NPs was 0.4 µg/mL for *FGF23* [54] (Figure 7a), a hormone regulating phosphate metabolism; for water‐PP NPs, it was 0.85 µg/mL for *ZNF274* [55, 56], associated with cancer and neurodevelopmental disorders. In vivo, the lowest BMDL was 4.8 mg/kg for *Ism2* [57], a gene linked to cardiac tissue integrity (Figure 7b). The BMDLs for cell viability after 24‐h exposure ranged from 4.83 µg/mL (oil‐PP NPs) to 111.8 µg/mL (water‐PP MPs)(Figure 7c). For in vivo phenotypic endpoints, the thresholds were 15.83 mg/kg for villus‐to‐crypt ratio reduction and 33.86 mg/kg for colon shortening in mice (Figure 7d).

To assess the relevance of these BMDL values, the safety thresholds, to real‐world conditions, we compared them with actual human exposure data using two complementary approaches. First, we estimated global intake levels of oil‐derived MNP based on dietary patterns and modeled 5‐year accumulation in the small intestine. Second, we compiled reported concentrations of PP MNPs in human biological tissues from previous studies, providing direct evidence of internal burdens across diverse populations.

We quantified per capita intake of PP MNPs across 23 representative regions spanning Asia, Europe, North America, South America, Africa, and Oceania using publicly available takeout food consumption data (Figure 7e; Table S6) and quantified the release of MNPs per container. The annual average intake of PP MNPs per adult ranged from 0.43 g in the United Kingdom to 3.35 g in China, with an average of 1.37 g per region (Figure S12g). Interestingly, the mean and median annual intake in developed countries were significantly lower than in developing countries (*p*<0.001; Figure S12h).

To translate these intake levels into in vivo tissue MNP burdens, we applied a one‐compartment input‐output model to estimate the accumulation of orally ingested oil‐PP particles in the small intestine across the same 23 regions shown in Figure 7e (Table S6). This modeling approach, widely used in pharmacokinetics and environmental risk assessment by the USEPA, assumes constant annual MNP intake, first‐order elimination with a 10‐year half‐life, and a homogeneous distribution within 1600 g [60] of intestinal tissue, based on ICRP reference values for adults [61]. We modeled a five‐year exposure period to reflect the recent global rise in food delivery and medium‐term chronic ingestion. For 200 nm particles (5% absorption), estimated cumulative intestinal concentrations ranged from 56 to 443 µg/g across regions. For 1 µm particles (0.1% absorption), concentrations remained below 9 µg/g. Using this model, we converted mouse gavage BMDLs into human‐equivalent oral intake BMDLs, which were then translated into five‐year tissue‐level safety thresholds for direct comparison with modeled intestinal MNP burdens (Table S7).

Surprisingly, all 23 regions exceeded the in vitro molecular and cell‐viability thresholds by at least one order of magnitude, with some regions reaching up to 1107‐fold (Figure 7f), indicating that real‐world dietary exposure to oil‐PP NPs may disrupt gene expression and impair cell viability. Notably, only the exposure level in China exceeded the in vivo molecular threshold, suggesting that more severe health effects, such as intestinal tight junction disruption or immune suppression, are more likely to occur in regions with high takeout food consumption. In contrast, none of the modeled burdens approached the BMDLs for tissue injury, suggesting that while subcellular disruptions likely occur at real‐world exposure levels, tissue‐level toxicity requires higher MNP exposures. For MPs, five‐year accumulations in all regions remained below the cellular viability BMDL for oil‐PP MPs (Table S6).

While in vivo studies capture organism‐level effects arising from high or cumulative exposures, in vitro assays provide more sensitive indicators of early cellular responses at lower exposure levels. Together, these models offer complementary perspectives on the continuum from molecular perturbation to tissue damage.

Next, to evaluate the actual accumulation of MNPs in humans, we compiled reported concentrations of PP MNPs from six types of human samples in previous studies [20, 21, 62, 63, 64, 65] (Table S8), assuming all exposures involved oil‐coated particles. Notably, even healthy placental samples exceeded the in vitro molecular thresholds for oil‐PP NPs (0.4 µg/mL) and water‐PP NPs (0.85 µg/mL), consistent with our previous findings [39] (Figure 7g). Moreover, higher concentrations in fecal and testicular samples even surpassed the cellular viability threshold for oil‐PP NPs (4.83 µg/g). These levels may disrupt cellular function and exacerbate pathological processes; however, none approached the modeled tissue‐level safety thresholds, again suggesting that overt tissue toxicity remains unlikely under typical dietary MNP exposure.

## 3 Discussion

Cooking oil, a common household ingredient, plays a critical yet often overlooked role in promoting micro‐ and nanoplastics migration from plastic food containers. Our results show that oil–plastic interactions, particularly during microwaving, markedly increase both MNP release and toxicity. Building on BMDLs derived from in vitro and in vivo toxicity data, we developed an integrated framework linking safety thresholds to real‐world exposure scenarios, revealing potential human health risks at the molecular and cellular levels. This work addresses critical knowledge gaps by revealing how routine practices, such as microwaving oil‐rich foods in plastic takeout containers, can elevate MNP exposure to biologically concerning levels.

Previous studies have suggested that oil facilitates the release of MNPs in plastic containers [9]. We confirmed and quantified oil's amplifying effect on MNP release, with increases of up to 125‐fold for PE‐coated containers and 29‐fold for PP containers, alongside co‐release of toxic chemicals and metals (up to 309‐fold), compared with water (Figure 2a,c). This enhanced release can be attributed to strong non‐polar interactions between oils and hydrophobic polymer chains, which allow oils to penetrate the polymer matrix more effectively than water. Such interactions, together with the wetting effect of lipids and potential thermo‐oxidative or other chemical reactions at the oil–plastic interface, increase additive mobility and accelerate thermal degradation, resulting in substantially higher levels of both MNPs and co‐migrating additives/metals under oil‐based conditions [43, 44]. More importantly, oil‐derived PP NPs displayed an oil‐film coating and a neutral to slightly positive surface charge, unlike the strongly negative charge of water‐derived particles (Figure 1g,i). This coating likely masks polar groups, stabilizes small fragments, and enhances tissue penetration, which can potentially persist during digestion due to hindered enzyme access and strong intermolecular interactions [66, 67]. Similar to the partial lipolysis and surface retention phenomena observed in lipid‐based self‐microemulsifying drug‐delivery systems [68]. In addition, the hydrophobic oil layer may lower the interfacial energy between PP surfaces and the phospholipid bilayer, making the nanoparticles more prone to associating with the cell membrane [69].

An important consideration in interpreting our toxicity data is that oil extraction releases not only more particles but also more chemical additives from the plastic matrix, as evidenced by our LC‐MS/MS analysis revealing numerous oil‐specific chemical features. These small molecules, undetected by our NanoSight particle counting, could contribute to the enhanced toxicity of oil‐PP NPs. The 4‐fold higher toxicity of oil‐PP NPs (IC_50_: 18.7 µg/mL) compared to water‐PP NPs (IC_50_: 77.1 µg/mL) likely reflects a combination of: (1) an oil‐specific shift in surface charge, as only soybean oil–derived PP NPs exhibited a transition to near‐neutral or positive zeta potential, which increases their propensity to adsorb serum proteins and form a protein corona, thereby enhancing receptor‐mediated cellular uptake in culture media [70]; (2) the oil film coating facilitating cellular uptake, which increases attraction to the phospholipid head‐groups of the cell membrane; (3) co‐extracted toxic additives; and (4) morphological differences, as oil‐PP NPs exhibited more irregular and fragmented structures (Figure 3g), which may increase membrane contact area compared to the smoother water‐PP NPs.

While we cannot yet definitively separate particle‐specific effects from those mediated by dissolved additives, the rapid membrane disruption observed within 5 min, together with the clear particle‐to‐cell contact seen under microscopy, indicates that oil‐modified nanoparticles possess intrinsic biological activity. At the same time, previous studies have shown that microplastics and nanoplastics often act together with their associated additives or co‐contaminants, resulting in additive or synergistic biological effects [71, 72, 73]. This evidence suggests that dissolved additives and other co‐released chemicals may also contribute to the toxicity observed in our system. To clarify their relative roles in future studies, approaches such as ultrafiltration or dialysis to separate nanoparticles from leachates, particle washing or surface stripping to remove loosely bound chemicals, and exposure to additive‐enriched extracts can be used to quantitatively distinguish particle‐specific from chemical‐specific effects. Regardless of the mechanism, our findings demonstrate that oil‐plastic interactions create a more hazardous exposure scenario than water‐plastic interactions, warranting regulatory attention.

Notably, the in vitro and in vivo immune transcriptional responses provided complementary insights into immunotoxicity (Figure S11d). HEK293T cells, lacking immune components, primarily show acute, cell‐autonomous responses to NPs exposure. In contrast, jejunal tissue from exposed mice showed predominant downregulation of immune genes, suggesting chronic immunosuppression or regulatory feedback from prolonged exposure. The alterations in immune‐related pathways observed in our study are consistent with intestinal barrier damage observed in vivo (Figure 5e,h). Loss of epithelial integrity can compromise mucosal immune signaling [74]. Specifically, pathways involved in T cell receptor, B cell, and NOD‐like receptor signaling were concurrently affected, highlighting a disturbance in intestinal immune responses.

In response to this disruption, our in vivo rescue experiments demonstrated that treatments with MDP (a NOD2 agonist targeting NOD‐like receptor signaling), NaB, and β‐Glucan partially restored immune function and epithelial repair (Figure 6c–f). These improvements were evidenced by enhanced villus‐crypt architecture, increased goblet cell density, and reduced epithelial apoptosis. Together, these findings emphasize the crucial role of restoring immune homeostasis in mitigating the effects of oil‐PP NP exposure. While we did not directly phenotype specific immune cell subsets, the affected pathways are highly enriched for signaling modules used by these cells, and the detailed immune‐cell profiling is an important direction for future work.

Taken together, our in vitro and in vivo data provide clear toxicity evidence at the molecular, cellular, and tissue levels. Using BMD modeling, we quantified safety thresholds for key endpoints across these levels (Figure 7a–d). The optimal BMD model was selected based on robustness, relevance, and sensitivity (Methods). We prioritized endpoints with strong responsiveness to nanoparticle exposure and critical biological pathways, which are essential for understanding the toxic effects of oil‐PP NPs on the gut and other organs. However, calculating the BMDLs is the first step toward linking MNP exposure to real‐life health risks. We therefore modeled the five‐year accumulation in intestinal tissues and collected data on real‐world tissue MNP burdens.

Oil‐derived MNPs release per container was quantified using absolute Py‐GC/MS and integrated with dietary intake patterns from 23 global regions to generate annual MNP intake data. These regions were selected based on the availability of consistent take‐out food consumption statistics, widespread reliance on disposable plastic food packaging, and the need to capture geographical and economic diversity across global dietary contexts. To translate these intake data into internal exposure levels, we applied a single‐compartment input–output accumulation model based on established toxicokinetic principles. This model provides a scientifically grounded and conservative estimate of MNP accumulation in the small intestine. To enhance transparency, we also acknowledge that future refinements could incorporate population variability (e.g., age‐, sex‐, and health‐dependent differences in gut physiology) and parameter‐uncertainty analyses to further improve individualized or demographic‐specific predictions. Over five years, predicted intestinal burdens exceeded molecular BMDLs by two to three orders of magnitude and cellular BMDLs by about one order of magnitude (Figure 7f), while remaining below the phenotypic BMDLs associated with organ‐ and tissue‐level effects.

Moreover, concentrations of PP MNPs measured in human biological samples, including placenta, feces, and diseased tissues, are comparable to or exceed the molecular and cytotoxic thresholds identified in our in vitro and in vivo data (Figure 7g). The agreement between modeled predictions and human measurements suggests that routine dietary habits, such as frequent consumption of oily takeout foods, may elevate internal MNP levels into biologically active ranges capable of triggering subclinical cellular stress, immune disruption, and early pathological processes.

Although current exposures may not cause overt tissue injury, the potential to drive chronic low‐grade inflammation, barrier dysfunction, and immune dysregulation should not be underestimated, which can possibly contribute to conditions such as irritable bowel syndrome (IBS), inflammatory bowel disease (IBD), and Crohn's disease. Our findings highlight a concerning alignment between established safety thresholds, modeled MNP intake levels, and reported internal MNP burdens in humans.

This study was conducted under controlled laboratory conditions with simplified simulants, standardized heating protocols, and a single‐compartment exposure model that assumes homogeneous intestinal distribution and first‐order clearance. Such conditions do not account for localized accumulation, mucus interactions, or segment‐specific transport, and direct clinical evidence linking oil‐derived MNP exposure to human health outcomes remains lacking. Future work should incorporate oil‐based simulants, size‐resolved exposure models, and sensitive molecular endpoints, alongside large‐scale epidemiological studies in populations with high intake of oil‐rich or reheated takeout foods. Evaluating alternative packaging materials with lower MNP release, such as bioplastics, glass, or stainless steel [75, 76, 77], and developing stable in vivo labeling methods for oil‐derived plastics will be critical for improving exposure assessment. Moreover, studies focusing on protein corona characterization and on specific uptake pathways will be essential to further elucidate the cellular mechanisms underlying oil‐derived nanoplastic toxicity.

Our study highlights the critical role of cooking oil in promoting the release and toxicity of smaller MNPs from PP‐ and PE‐coated containers during microwave heating, findings supported by both in vitro cytotoxicity and in vivo intestinal injury in mice. Alarmingly, the estimated MNP burdens in the small intestine across 23 global regions and the reported MNP levels in human tissues all exceeded the molecular and cellular viability safety thresholds, suggesting that routine dietary intake may be sufficient to trigger adverse cellular responses. These findings raise significant concerns about chronic dietary exposure to oil‐derived MNPs and associated contaminants, calling for stricter regulations on plastic food packaging for oil‐rich foods and developing safer alternatives.

## 4 Methods

### 4.1 Characterizations of Plastic Containers

Two anonymous brands of plastic food containers made of PP and PE materials were purchased from online stores (sales exceeding 3 million units) and restaurants in Hangzhou, China. These two types of containers were selected due to their widespread use in food packaging and delivery services.

The compositions of the plastic containers were determined by the Attenuated Total Reflectance Fourier Transform Infrared spectroscopy (ATR‐FTIR, NICOLET iS50FT‐IR, Thermo Scientific, USA). The obtained IR spectra were compared with the database in OMNIC software, and a polymer type was considered acceptable when the match with the standard spectra was greater than 70%. At least three different batches of the containers were purchased at different times of the year and analyzed in the study.

Their semicrystalline structure and thermal stability were analyzed by differential scanning calorimetry (DSC) using a Q200 differential scanning calorimeter (TA Instruments, New Castle, DE, USA). Approximately 8 mg sample was taken from each container, placed in an aluminum pan, sealed, and subjected to a thermal cycle at 10°C/min under a nitrogen atmosphere. The resulting calorimetric curves, which reflect heat transfer during the thermal cycle, were used to monitor phase transitions.

X‐ray diffraction (XRD) has been used to detect changes in the crystalline and amorphous characteristics of plastic materials. To prepare the samples, we cut the PP containers and the inner plastic lining of the PE‐coated containers into 0.5 × 0.5 cm pieces. Specimens were kept in an aluminum sample holder so that the upper surface was smooth and exposed to X‐rays in the vertical goniometry assembly. The scan was taken between (10°–80°) 2θ with a scanning speed of 0.02‐degree 2θ per min.; the operating target voltage was 35 kV, tube current was 20 mA, and radiation used was FeKα with a wavelength of 1.93735 Å on Rigaku Rotating anode mode RU‐H3R (18Kw), X‐ray powder diffractometer. The intensity versus 2θ scans were obtained for these plastic materials.

A high‐resolution field‐emission scanning electron microscope (SEM, Regulus 8230, Hitachi, Japan) was used to examine the surface morphology of the inner walls of the plastic containers before and after treatment.

### 4.2 Release Experiments of Micro‐ and Nanoplastics

In total, three common real‐life takeout food scenarios using plastic containers were simulated: microwaving food (Groups A‐C), transportation (Group D‐F), and leftover storage (Groups G and H). To simulate different food types, food simulants were used: ultrapure deionized water (DI water; 18.2 MΩ·cm, Cascada water purification system, Pall, USA) and commercial cooking oils of anonymous brands (soybean, palm, blend, peanut, and sunflower oil) purchased from brand stores in China. DI water and cooking oils were stored in glass beakers and analyzed separately. Prior to the experiments, the PP and PE‐coated containers were thoroughly rinsed with DI water 3 times and air‐dried to remove residual MNPs from the manufacturing process.

For all experiments, containers were filled with 100 mL of a single oil type or DI water (Figure S1a). To simulate microwaving, containers filled with food simulants at room temperature were placed in a microwave oven (M1‐201A, Midea, China) at 800 W for 1, 3, and 5 min. Following the previous study [78], containers filled with food simulants were oscillated at 120 rpm on a horizontal rotary shaker (NMSP‐600, NuoMi, China) for transportation simulation at room temperature for 15, 30, and 60 min. For the leftover storage simulation, the containers were filled with oil or water, pre‐heated to 95°C, and left at room temperature for 1 and 5 h. We modified a previous study's procedure to concentrate the leachate and extract the plastic particles for subsequent analysis [13]. For oil‐treated samples, 100 mL of oil was mixed with 300 mL of hexane (1:3 ratio) and vortexed for 30 s to ensure complete dissolution. Using a vacuum pump, the mixture was filtered through a vacuum filtration system. To remove residual oil, 60 mL of prefiltered hexane was added to the Anodisc filters (0.22 µm pore diameter, 25 mm diameter, Waterman, Germany) three times to wash the particles. Water‐treated samples were poured directly into the vacuum filtration system for extraction. The filter membranes from both oil‐ and water‐treated samples were then placed in a glass dish filled with 10 mL of 30% H_2_O_2_ and incubated at 60°C for 1 h to remove excess organic compounds. The filters were subsequently sonicated for 1 h to yield 10 mL concentrate leachate samples of MNPs for downstream analysis.

### 4.3 Characterization of Micro‐ and Nanoplastics

Optical microscope (LV100N, NIKON, Japan), SEM, and Transmission Electron Microscopy (TEM, JEM‐1400flash, JEOL Co., Ltd., Japan) were used to characterize the morphology of MPs and NPs.

Leachate samples (100 mL) from each experimental group were filtered through a 0.22 µm glass fiber filter (Shanghai Dibo Biotechnology Co., Ltd., Shanghai, China). Plastic type and chemical composition were determined by ATR‐FTIR spectroscopy (NICOLET iS50 FT‐IR, Thermo Scientific Inc., Waltham, MA).

Zeta potential was measured using a laser particle sizer (Zetasizer Nano ZSE, Malvern, UK) with 1 mL of MNP suspension placed directly into the measurement cell.

MNPs were characterized by Raman microspectroscopy (Horiba Scientific, Kyoto, Japan) using soybean oil–treated samples, assuming that water‐treated samples had comparable properties. Analyses were performed on a Raman XploRA Nano Microspectrometer equipped with a 785 nm laser and 600 lines mm^−^^1^ grating (0–2200 cm^−^^1^ spectral range). Calibration was conducted with the silicon reference peak at 520.7 cm^−^^1^. Spectra were processed via polynomial baseline correction and vector normalization in LabSpec 6. Identification was achieved by matching spectra to the SLOPP Library of Microplastics and KnowItAll software (Bio‐Rad Laboratories, Inc.), with a Hit Quality Index (HQI) ≥ 80 considered reliable.

### 4.4 Micro‐ and Nanoplastics Detection and Quantification

We followed our previous protocol to assess the size and quantity of microplastics in the leachate [39]. A 5 µL droplet of the 10 mL concentrated leachate was mixed with 195 µL of 0.01 mg/mL Nile Red (Aladdin, China) solution in a 96‐well plate, and the mixture was incubated at 55°C for 30 min for staining. Each leachate sample was processed in triplicate to ensure statistical robustness, with three blank controls included per plate. Fluorescence was measured using a BioTek Cytation 3 plate reader (BioTek Instruments, Inc., USA) at 4x magnification, with the Montage function used to quantify MPs as small as 6 µm in diameter. Consistent acquisition and analysis parameters were applied to all wells.

To assess the size and quantity of nanoplastics present in the leachate samplers, we used the Nanoparticle Tracking Analysis (NTA, NanoSight NS500, Malvern Panalytical Ltd, UK), which is equipped with a 532 nm green laser to detect and count particles in the 10 nm to 1 µm size range, as described in a previous study [79]. Three leachate samples were analyzed for each release experiment. We note that NTA has a practical detection limit of approximately 10 nm, meaning smaller particles and dissolved materials are not captured in our particle count data. Therefore, our reported particle concentrations (particles/L) represent only the fraction of particles >10 nm.

For toxicity assessments, we used mass concentration (µg/mL) based on Py‐GC/MS to ensure accurate dosing, though we cannot exclude the contribution of sub‐10 nm particles or dissolved polymer chains to the observed effects. We selected 3‐min microwaving samples for Py‐GC/MS analysis. The samples were filtered through a 25 mm GF/F glass fiber filter (220 nm mesh, Whatman, UK). To eliminate plastic contamination, the filters were preheated in a 500°C nitrogen‐purged muffle oven before filtration. Plastic particles were retained on the filter. The residue was then rinsed with 10 mL of 30% H_2_O_2_ (Merck, Germany) and 15 mL of deionized (DI) water. The inner circle containing the analyte (Figure S2e) was cut from the filter using a stainless‐steel blade, dried at 45°C for 4 h, and subsequently transferred to a pyrolysis cup. Analysis was performed using the multishot pyrolysis unit EGA/PY‐3030D (Frontier Laboratories, Saikon, Japan) in “single shot” mode. The GC/MS system (Trace 1300 GC and ISQ 7000 MS, Thermo Fisher, Waltham, MA, USA) was equipped with a TG‐5SILMS column (30 m × 0.25 mm × 0.25 µm, Thermo Scientific Inc., Waltham, MA, USA). The temperature program started at 40°C, held for 2 min, then increased at a rate of 20°C/min to 320°C and held at 320°C for 14 min. Measurements were conducted in selected ion monitoring (SIM) mode with a 1:50 split ratio. During the analysis, an ion scan range of m/z 29–600 was used to identify and quantify the polymers of the target plastic particles. Indicator ions for PP and PE, 2,4‐dimethyl‐1‐heptene (m/z 126) and 1‐docosane (m/z 83), were employed. Standards for target microplastics (PP and PE) were first analyzed, and standard curves for PP and PE quantification were constructed to determine the mass of MNPs in all samples (Figure S3e and Table S1). The NIST17 library spectrum was used for compound identification, and peak areas were calculated using the normalization method.

### 4.5 Heavy Metal Analysis

Following established protocols [80], the samples were homogenized and digested using a microwave digestion system (Anton Paar, Graz, Austria) with a tri‐acid mixture of nitric acid (HNO_3_), sulfuric acid (H_2_SO_4_), and perchloric acid (HClO_4_) in a 5:1:1 ratio. Post‐digestion, the samples were filtered, diluted in ultrapure water, and analyzed using an Inductively Coupled Plasma Mass Spectrophotometer (ICP‐MS) (Agilent Technologies 7800 ICP‐MS, Santa Clara, CA, USA).

### 4.6 Analysis of Organic Additives in Plastic Materials

We followed the protocol of the previous study [81]. Methanol (99.8%, Sigma‐Aldrich) was used to extract chemicals from plastic materials, as it solubilizes a wide range of compounds without dissolving the polymers. To minimize contamination, all consumables, except pipette tips, were made of glass or stainless steel, rinsed with ultrapure water and acetone, and heated at 200°C for at least 2 h. Each 1.5 g sample was cut into small pieces (0.5–0.8 × 2 cm; thickness ≤ 0.4 cm) and extracted with 9 mL methanol in glass vials with polytetrafluoroethylene‐lined lids. Extraction was performed by sonication for 1 h at room temperature. One milliliter of extract was collected for chemical analysis and stored in glass vials at −20°C. Four procedural blanks (PB1–4) containing only methanol were processed in parallel to monitor potential contamination.

Samples were subjected to nontargeted LC‐MS/MS by an Acquity UPLC BEH C18 column (2.1 × 100 mm i.d., 1.7 µm, Waters) coupled to a SCIEX X500B quadrupole time‐of‐flight (Q‐TOF) mass spectrometer (AB SCIEX Pte. Ltd., USA) in positive ionization modes. Data acquisition was performed in full MS scan mode and in information‐dependent acquisition (IDA)‐MS2 scan mode. During data acquisition, the quality control (QC) sample (generated by pooling all the samples) and blank samples (laboratory, transport, field, and extraction) were injected between every 10 sample injections.

Mass spectrometry data were centroided and converted from the proprietary format (.raw) to m/z extensible markup language (.mzML) using ProteoWizard MSConvert (v3.0.21094). Following a comparison of upstream processing parameters using IPO, Autotuner, and SLAW, SLAW was selected for parameter optimization, mass feature and isotopic pattern extraction, peak grouping, and deisotoping. The resulting feature quantification tables (.csv) and MS^2^ consensus spectra (.mgf) were exported for further analysis. Spectra from each sample were processed independently to avoid inaccuracies arising from joint retention‐time alignment, given their differing chemical compositions. Features (ions with unique m/z and retention time) with abundances <10‐fold the maximum detected in PBs and solvents were excluded. Remaining feature abundances were corrected by subtracting the maximum abundance of the corresponding feature observed in PBs.

For data annotation, metabolite identification was performed using spectral entropy based on accurate mass (±0.01 Da) and MS^2^ similarity to public databases. Specifically, the MS^2^ reference database was constructed by integrating open‐source databases from the GNPS community (495 662 spectra as of 2022‐06‐18), the MoNA dataset (1 951 233 spectra as of 2023‐02‐07), and the NIST2020 Tandem Mass Spectral Library (1 143 815 spectra). All metabolites annotated through spectral matching were considered level 2 annotations.

### 4.7 In Vitro Cell Viability Study

We used a 4‐h high‐concentration exposure (50 and 100 µg/mL) to model acute, high‐dose intake events, such as ingestion of highly contaminated food or water during the typical postprandial digestion and absorption period when PP NPs interact with gastrointestinal cells and translocate into the systemic circulation. Conversely, we used a 20‐h low‐concentration exposure (10 and 25 µg/mL) to model cumulative retention from repeated dietary intake throughout the day, mimicking gradual nanoparticle buildup in tissues and digestive compartments.

To assess the potential toxicity of the oil film, we first estimated the volume of oil coating the surface of PP NPs. A total of 250 PP NPs were analyzed from at least five TEM images using ImageJ software (National Institutes of Health, USA), comprising 150 oil‐coated particles and 100 uncoated particles. The average particle size was measured separately for oil‐coated and uncoated PP NPs. For volume estimation, nanoparticles were assumed to be uniform, non‐deformable spheres with a well‐defined core and an oil‐coated outer layer. This allowed an estimation of the oil film thickness and calculation of the total oil volume in the suspension (Table S4). The oil film volume was determined using the following equations:

$$V_{oil,\;single\;}=\frac{4}{3}\pi \;\left[{\left(\frac{D_{total}}{2}\right)}^{3}-\;{\left(\frac{D_{core}}{2}\right)}^{3}\right]$$

here, *V*_*oil*, *single* _ represents the oil film volume per nanoparticle, *D_total_* is the total diameter of the oil‐coated PP NP (measured from TEM images, 150.95 nm), and *D_core_* is the diameter of the PP NP core without the oil film (63.68 nm). By subtracting the core volume from the total volume, we obtained the volume of the oil layer surrounding each nanoparticle.

To estimate the total oil film volume in the nanoparticle suspension, we used a formula that incorporates the nanoparticle mass concentration, the density of polypropylene, and the single‐particle oil film volume:

$$V_{oil,\;total}=\frac{C_{mass}}{\frac{4}{3}\pi {\left(\frac{D_{core}}{2}\right)}^{3}\times \;\rho_{PP}}\times \;V_{oil,\;single}$$

here, *V*_*oil*, *total*_ is the total oil film volume in the solution, *C_mass_* is the PP NP mass concentration (µg/mL), and ρ_*PP*_ is the density of polypropylene (∼0.91 g/cm^3^). The denominator represents the estimated number of nanoparticles per µL of solution, calculated from the core volume and material density.

These estimations provide an approximation of oil exposure but do not account for potential aggregation of nanoparticles, variations in oil adsorption efficiency, or dynamic changes in oil film properties over time. The actual bioavailability and toxicity of the oil film may also be influenced by interactions with environmental and biological factors, including surfactant behavior, protein corona formation, and enzymatic degradation.

#### 4.7.1 Preparation and Characterization of Micro‐ and Nanoplastics from PP Containers

Polypropylene (PP) food containers, among the most widely used microwaveable plastics, were selected for cytotoxicity assessment. Twenty grams of PP plastic were placed in an autoclaved glass bottle containing 200 mL of deionized (DI) water or cooking oil, and the bottle was microwaved at 800 W for 3 min to simulate domestic heating.

Water‐PP MPs / Oil‐PP MPs: MNPs suspensions were filtered through a PTFE membrane (0.8 µm; Jingteng, China). Retained particles were rinsed into a glass dish with ethanol, vortexed for 30 s, and centrifuged at 15 000×g, 4°C for 10 min.

Water‐PP NPs: Suspensions were filtered through a PTFE membrane (0.2 µm); filtrates were concentrated using a 20 nm Anodisc membrane, rinsed with ethanol, vortexed for 30 s, and centrifuged at 15 000×g, 4°C for 10 min.

Oil‐PP NPs: Suspensions were filtered through a PTFE membrane (0.2 µm), and the filtrates were centrifuged at 15 000×g, 4°C for 20 min.

The isolated particles were either used directly for physicochemical characterization or subjected to further purification prior to cellular assays.

Pellets were resuspended in ethanol, ultrasonicated for 10 min, and centrifuged at 15 000×g, 4°C for 20 min, followed by three cycles of resuspension in 0.1% SDS, ultrasonication for 10 min, and centrifugation. The final pellet was resuspended in 0.1% SDS, ultrasonicated for 30 min to ensure homogeneous dispersion, sterilized, and stored. A control containing only 0.1% SDS (no particles) was included to account for surfactant effects.

Particle morphology was examined by transmission electron microscopy (TEM). Fluorescently labeled oil‐PP NPs were visualized using a super‐resolution microscope (GE DeltaVision OMX SR, GE Healthcare, USA) with a 588 nm laser. Dynamic light scattering (Zetasizer Nano ZSE, Malvern, UK) was used to measure particle size distribution, zeta potential, and polydispersity index (PDI) after 30 min ultrasonication; nanoparticle tracking analysis (NTA) was also performed.

#### 4.7.2 Cytotoxicity Assay

Human embryonic kidney 293T (HEK293T) cells were maintained in RPMI 1640 medium, while human colon adenocarcinoma (HT29) and Caucasian colon adenocarcinoma (Caco‐2) cells were cultured in DMEM supplemented with 10% fetal bovine serum and 1× penicillin/streptomycin. All cells were incubated at 37°C in a humidified atmosphere containing 5% CO_2_.

For the CCK‐8 assay, HEK293T cells were seeded in 48‐well plates (5 × 10^4^ cells/well) and incubated for 24 h, then exposed to PP MPs or NPs (2–2000 µg/mL) for 24 h. Twenty microliters of CCK‐8 solution were added per well, and absorbance was measured at 450 nm.

For LDH release assays, cells were seeded in 48‐well plates (5–10 × 10^5^ cells/well) and exposed to NPs for 3 h. Supernatants were collected, and LDH activity (mU/mL) was measured using a commercial kit according to the manufacturer's instructions. All assays were performed in triplicate, and results were normalized to controls containing culture medium with 0.1% SDS only.

#### 4.7.3 Investigating the Cellular Uptake of NPs

For fluorescence labeling, oil‐PP NPs and water‐PP NPs were labeled with iDye Poly Pink (Rupert, Gibbon & Spider, Inc., Healdsburg, CA, USA). 0.01 g of the dye was combined with 1 mL of the stock particle suspension and incubated at 70°C for 2 h. After cooling down to 25°C, the mixture was diluted with 9 mL of ethanol and centrifuged at 4000 rpm for 15 min. This washing process was repeated twice. The fluorescence‐labeled PP NPs were then collected and resuspended in 1 mL of 0.1% SDS solution.

HEK293T cells were inoculated in a 6‐well plate. The cells were incubated with fluorescent NPs (FL‐oil‐PP NPs or FL‐water‐PP NPs) at a 100 µg/mL concentration for 0‐60 min. The cells were washed twice with PBS (1×) at room temperature, and they were treated with PBS (1×) containing 0.1 % Triton X‐100 for 3–5 min to make the membrane more permeable. To reduce background nonspecific staining, 1 mL of PBS (1×) containing 1% bovine serum albumin was incubated with the cells for 30 min. Finally, the cell nuclei were counterstained with Hoechst 33342. Fluorescence images were obtained using a laser confocal microscope (ECLIPSE TI, NIKON, Japan). The experiment was conducted in the dark.

#### 4.7.4 TEM Analysis

The cell microstructure and integrity were investigated using TEM (JEM‐1400 Flash, JEOL Ltd., Tokyo, Japan). After 5 min of exposure to 100 µg/mL NPs, the cells were fixed in 2.5% glutaraldehyde at 4°C for 30 min. The fixed cells were washed, treated with osmium tetroxide, and dehydrated using ethanol solutions. Ultrathin cell sections (70–90 nm) were obtained and analyzed by TEM according to a previously published protocol [46].

### 4.8 In Vitro Simulated Gastrointestinal Digestion of Oil‐PP MNPs

The simulated digestive fluids of the oral, stomach, and small intestine were prepared according to a published study [82]. Oil‐PP MNPs were separately digested by the simulated oral, stomach, and intestinal fluids. Briefly, 250 µg oil‐PP MNPs were spiked into 16 mL of artificial saliva and incubated for 5 min. Subsequently, 6 mL of digested saliva was added to 14 mL of gastric juice, and the pH was adjusted to 2.2 with 2 M HCl. The solution was incubated for 2 h. Then, 10 mL of the digested fluid was mixed with 10 mL of intestinal juice, and the pH was adjusted to 7.5 with 2 M NaOH. The solution was incubated for 2 h. Note that all digestions were incubated at 37 °C and shaken at 125 rpm. Enzymes were added to the electrolyte solution after pH adjustment, and the pH was checked again before incubation. To prevent interference from large, poorly soluble components or solid impurities, as‐prepared digestive fluids were filtered through a 5‐µm cellulose acetate syringe filter (Merck Millipore, Darmstadt, Germany) before each incubation.

### 4.9 In Vivo Toxicity Study

The animal experiments were conducted in accordance with Zhejiang University's Institutional Animal Care and Use Committee protocols (Approval number: ZJU20250612). We used 36 male, 4‐week‐old, specific‐pathogen‐free C57BL/6 J mice, sourced from Zhejiang University's Laboratory Animal Center and maintained under optimal conditions, including a temperature of 21 ± 3 °C, a humidity of 50 %, and a 12‐h light‐dark cycle. After a week of acclimatization, mice were randomly assigned to three groups (n = 12 per group) and standardized by body weight to begin the experiment uniformly. Mice from all three experimental groups were randomly allocated across all cage positions to control for any confounding effects of cage location.

To simulate chronic dietary exposure to oil‐derived nanoplastics from takeout food, oil‐PP NPs (≤200 nm) were extracted from microwave‐heated soybean oil and suspended in 1% Tween 80. Male C57BL/6J mice (∼25 g) were randomly assigned to control, low‐dose (10 mg/kg), or high‐dose (50 mg/kg) groups, and administered 200 µL of oil‐PP NPs suspension via oral gavage every three days on days 0, 3, 6, 9, 12, and 15. Our two‐week gavage regimen captures only subacute effects and cannot fully model truly chronic, lifelong dietary exposure. Because plastic particles can bioaccumulate and elicit low‐grade, progressive damage over months or years, longer studies with extended dosing intervals (e.g., 3–6 months) will be necessary to assess cumulative tissue burdens, adaptive or compensatory responses, and potential recovery dynamics.

Doses were derived based on two complementary approaches. First, a human‐to‐mouse body surface area normalization method recommended by the U.S. FDA was applied to extrapolate daily human exposure into murine equivalents [83]:

$$Mouse\;dose\left(\frac{mg}{kg}\right)=Human\;dose\left(\frac{mg}{kg}\right)\times \left(\frac{Human\;K_{m}\;}{Mouse\;K_{m}}\right)$$

where *K_m_* represents the species‐specific BSA normalization coefficient (37 for humans and 3 for mice), yielding a conversion factor of 12.33. Second, a direct exposure‐based estimation was conducted using experimentally measured nanoplastic release data. Py‐GC/MS analysis showed that microwaving a single oil‐filled polypropylene (PP) container released approximately 39.11 mg of oil‐PP MNPs, predominantly ≤200 nm in size. For a 60 kg adult, this corresponds to an intake of 0.652 mg/kg per container, while ingestion from six containers would equal 3.91 mg/kg.

Applying FDA‐recommended BSA scaling, a human intake equivalent to one container every three days corresponds to approximately 8.04 mg/kg in mice, whereas six containers over the same period correspond to 48.21 mg/kg. Based on this calculated range, 10 mg/kg and 50 mg/kg were selected as the low‐ and high‐dose levels, respectively. The 10 mg/kg dose approximates exposure from approximately 1 oil‐containing takeout container over 3 days, reflecting a realistic consumption pattern of approximately one takeout meal every 3 days. The 50 mg/kg dose represents a high‐exposure scenario equivalent to about 6 containers over three days, corresponding to frequent takeout consumption (approximately 2–3 meals every three days). Together, these doses bracket a human‐relevant exposure range while enabling evaluation of dose‐dependent intestinal effects.

We acknowledge that quantitative extrapolation of these doses to humans is subject to inherent uncertainties, including differences in toxicokinetics, interindividual variability in dietary habits, and the metabolic handling of oil‐associated plastic particles and additives. Therefore, the applied in vivo doses should be interpreted as mechanistically relevant intake scenarios rather than precise human exposure equivalents.

### 4.10 MDP, NaB, and β‐Glucan Intervention in Oil‐PP NP–Exposed Mice

Male C57BL/6J mice were randomly divided into six groups: control, oil‐only, oil‐PP NPs, and three intervention groups (oil‐PP NPs+MDP, oil‐PP NPs+NaB, and oil‐PP NPs+β‐glucan; n = 6 per group, except the oil‐only group, n = 5). Oil‐PP NPs (≤200 nm) were isolated from microwave‐heated soybean oil and resuspended in 1% (v/v) Tween‐80. Mice in the oil‐PP NP groups received 200 µL of oil‐PP NP suspension (25 mg kg^−^^1^) by oral gavage every 3 days for 14 days. The oil‐only group was gavaged with 200 µL of microwaved soybean oil prepared under identical conditions, but without plastic containers.

MDP, NaB, and β‐glucan were dissolved in 0.5% (w/v) sodium carboxymethyl cellulose (CMC‐Na) and administered once daily by oral gavage at a volume of 100 µL per mouse, at the following doses: MDP at 150 µg/mouse, NaB and β‐glucan at 200 mg kg^−^^1^ per day. Treatments began 3 days before the first oil‐PP NP exposure and continued throughout the exposure period. Control and oil‐PP NP groups received the corresponding vehicle (100 µL of 0.5% CMC‐Na without drug) on the same schedule.

### 4.11 Histological Analysis

The jejunal segments (∼1–2 cm) were excised, rinsed with cold PBS to remove luminal contents, and fixed in 4% paraformaldehyde at 4°C overnight. Tissues were then dehydrated, embedded in paraffin, and sectioned at 4 µm for histopathological evaluation, including hematoxylin and eosin (H&E) staining, Alcian blue–periodic acid–Schiff (AB–PAS) staining for goblet cells, and TUNEL assays for apoptotic epithelial cells (n ≥ 5 per group). H&E staining was used to assess overall mucosal architecture and villus–crypt morphology, AB–PAS staining to quantify goblet cell density, and TUNEL to calculate the proportion of TUNEL‐positive epithelial nuclei. Quantitative analysis of villus length‐to‐crypt depth ratio, goblet cell density, and TUNEL‐positive cell ratio was performed using ImageJ under identical imaging conditions across groups.

### 4.12 Transcriptomic Analysis

The jejunal samples were snap‐frozen in liquid nitrogen and stored at −80 °C for RNA sequencing (n = 6 per group). The RNA sequencing and library construction were performed by Annoroad Gene Technology Co., Ltd. (Beijing, China) and Novogene Co., Ltd. (Beijing, China). Kallisto software (v0.46.1) [84] was used to quantify transcript abundance from RNA‐seq data against the GRCh38 (human) and GRCm39 (mouse) cDNA reference transcriptomes from the Ensembl database. The Tximport package (v1.32.0) [85] integrated transcript‐level estimates into gene‐level expression matrices using the TxDb.Hsapiens.UCSC.hg38.knownGene (v3.18.0) [86] and TxDb.Mmusculus.UCSC.mm39.knownGene (v3.18.0) databases, respectively. Gene differential expression analysis and data normalization were performed with the DESeq2 package (v1.44.0) [87]. Functional enrichment analyses were conducted using ReporterScore (v0.1.8) [88] together with org.Hs.eg.db (v3.19.1) [89] for human data and org.Mm.eg.db (v3.19.1) for mouse data.

### 4.13 Human MNP Exposure Estimation

We estimated the human exposure to MNPs and heavy metals using MNP and heavy metal release rates from PP and PE‐coated containers filled with oil under 3‐min microwave heating (data from this study), the frequency of people ordering takeout food, and the adults (18‐59 years old) and kids (0‐3 years old) daily fat intake mass (Table S5). We also collected data on the frequency of takeout food orders across 23 regions worldwide to calculate the global exposure to MNPs (Table S6). These regions included in this analysis were selected because they represent major global populations, cover a wide range of socioeconomic and dietary patterns, and provide a suitable framework for standardized cross‐regional comparison. The takeout frequency data used for these regions were obtained from Statista, YouGov, Yandex, and Opinion Box. The MNP and heavy metal exposure were assessed using the following equation:

$$MHP_{i}=V_{i}\times mhp_{Ii}\times F_{j}$$

Here, “*MHP_i_*” is the annual intake of MNPs or heavy metals (grams per year), index “i” refers to the type of plastic containers (PP container and PE‐coated container), “*V_i_*” is the intake weight of the average meal (fat) per person obtained from Food and Agriculture Organization of the United Nations (FAO) [90, 91], we used lunch for the annual intake calculation of MNPs and heavy metals, accounting for 40% of the total daily food intake, based on the dietary ratio of 30%:40%:30% for breakfast, lunch, and dinner, respectively (Table S5). Lunch was selected because it typically represents the largest single meal, is most frequently associated with takeout container use in many regions, and provides a standardized, conservative exposure scenario for cross‐country comparisons. “*mhp_Ii_*” is the concentration of MNPs and heavy metals that migrated into oil under 3‐min microwave heating (e.g., MNPs released from PP container filled with oil: 0.43 g g^−1^, MNPs released from PE‐coated container filled with oil: 1.68 g g^−1^), index “j” refers to the frequency of consuming take‐out food, and “*F_j_*”, is the total number of takeout food orders a person consumes annually based on the different frequencies of take‐out food orders. It is important to note that this calculation only estimates the amount of MNPs and heavy metals ingested through dietary exposure and does not account for potential digestion, degradation, or transformation of MNPs in the gastrointestinal tract. The actual bioavailability and toxicity of ingested MNPs may vary depending on their physicochemical properties and interactions with digestive processes.

We estimated the five‐year accumulation of oil‐PP MNPs in human small‐intestine tissue using a simplified one‐compartment input–output model. Calculations proceeded in three steps: annual input rate (*R_in_*), elimination rate constant (*k_e_*), and five‐year accumulated concentration (*C*_5_).

Annual Input Rate (*R_in_*)

$$R_{in}=\frac{M_{year}\times 10^{6}\times F_{gut}}{V_{gut}}\;\left(\mu gg^{-1}year^{-1}\right)$$

Five‐Year Accumulation (*C*_5_)

$$K_{e}=\frac{\ln2}{t_{1/2}}\left(year^{-1}\right)$$

$$\frac{dC}{dt}=R_{in}-k_{e}\left(1-e^{-k_{e}t}\right)$$

With *C*(0) = 0, the solution is

$$C\left(t\right)=\frac{R_{in}}{k_{e}}\left(1-e^{-k_{e}t}\right)$$

Thus, at *t* = 5 years,

$$C_{5}=\frac{R_{in}}{k_{e}}\left(1-e^{-k_{e}\times 5}\right)\;\left(\mu g\;g^{-1}\right)$$

Here, “*M_year_*” is the annual mass of oil‐PP MNPs ingested (grams per year). “*V_gut_*” denotes the total mass of small‐intestine tissue (wall + contents) per person, taken here as 1600 g (≈1.6 kg) based on ICRP Publication 89^60^. “*F_gut_*” is the fraction of ingested MNPs that translocate from the gut lumen into the intestinal tissue, $F_{gut}^{200}$ = 0.05 (5 %) interpolated from Caco‐2 and rodent gavage studies reporting 2–14 % absorption [92]; For 1 µm microparticles, $F_{gut}^{1000}$ = 0.001 (01 %) reflecting experimental observations of <1 % uptake for particles >1 µm. “$t_{1/2}$” is the elimination half‐life of MNPs in small‐intestine tissue, here conservatively assumed to be 10 years to represent chronic retention. “*k_e_*” is the first‐order elimination rate constant for MNPs in the tissue. It defines the fraction of the tissue concentration that is cleared per unit time and is related to the elimination half‐life “$t_{1/2}$”. These definitions estimate only the mass of MNPs entering intestinal tissue; they do not account for gastrointestinal degradation, particle transformation, or variable bioavailability, all of which may influence actual systemic exposure and toxicity.

We systematically compiled all available Py‐GC/MS–based quantitative measurements of microplastics reported in human organs (Table S8). In subsequent analyses, only tissues with explicitly quantified PP concentrations were considered. This conservative strategy ensures that risk contextualization relies on polymer‐specific, mass‐based PP data rather than aggregated MNP burdens, thereby preserving consistency with the PP‐focused framework adopted in this study and minimizing uncertainty associated with mixed‐polymer measurements.

To evaluate toxicological responses, benchmark dose (BMD) calculations were performed using data with significant dose‐response relationships. This included both cell viability data and molecular markers, as illustrated in Figures 3a and 6d. We employed a continuous endpoint approach for all features. The selection of the optimal BMD model within the USEPA BMDS framework was conducted through a consensus‐based approach. This involved multiple criteria, including a goodness‐of‐fit threshold with a *p*‐value greater than 0.10; the lowest AIC; a BMD/BMDL (lower 95 % confidence interval) ratio less than 5; and a visual inspection of the curve fit to ensure both plausibility and model parsimony. For our analysis, the default confidence level was set at 95 %, corresponding to a one‐sided 95 % confidence limit. We opted to use the lower confidence limit (BMDL) to derive health guidance values, as it provides a more conservative, precautionary estimate of the toxic dose. For gene‐based BMD analysis, we used the BMDExpress software (v2.30.0515 BETA) to calculate the transcriptomic‐level BMD and BMDL.

### 4.14 Quality Assurance and Control (QA/QC)

To minimize potential plastic contamination, multiple measures were implemented throughout the experimental process. Plastic containers used for the experiments were carefully selected from the middle of each stack to avoid contamination from external plastic particles or debris. Laboratory personnel wore nontextile lab coats and particle‐free nitrile gloves to prevent secondary contamination, with lab coat sleeves securely tucked into gloves at all times. Additionally, all work was conducted in a clean workspace with minimized airflow disturbances to reduce the risk of airborne particle contamination.

All glassware used in the experiments was rigorously cleaned by rinsing three times with anhydrous ethanol, followed by ultrapure water, to ensure no residual contaminants remained. Ultrapure water and ethanol were filtered to remove any possible plastic contaminants before use. Equipment and work surfaces were thoroughly cleaned and inspected before each experiment.

To ensure reproducibility and reliability, each experiment was conducted in at least triplicate using three identical containers per condition. The results from these replicates were averaged to account for variability and reduce random errors. Blank controls (no plastic container or sample added) were also included in each experimental setup to monitor and quantify background contamination levels, ensuring that any observed results originated solely from the experimental treatments.

For MP and NP detection and quantification, rigorous quality control measures were implemented. The fluorescence measurements were calibrated using certified polystyrene microsphere standards (1–100 µm), maintaining RSDs below 10%. NTA analysis included daily calibration with NIST‐traceable standards (100 and 200 nm), with measurements accepted only when standard values were within ±5% of nominal values. For Py‐GC/MS analysis, method blanks were run every 10 samples, with CCVs analyzed every 12 h. Method detection limits were 0.06 mg/mL for PP and 0.09 mg/mL for PE, with recoveries of 96% and 123%, respectively. And calibration curves maintaining R^2^ > 0.99.

For metal analysis by ICP‐MS, quality control included analysis of certified reference materials (NIST SRM 1643f) every 20 samples. The instrument was calibrated using multi‐element standard solutions (0.1–100 ppb), with internal standards (Sc, In, Bi) used to correct for matrix effects and instrument drift. Method blanks were analyzed every 10 samples, and detection limits were set at 3σ of the method blanks. Recovery rates for spiked samples ranged from 85%–115%, with RSDs < 10% for all elements. Duplicate analyses were performed every 10 samples, with acceptance criteria of ±15% relative percent difference.

For LC‐MS/MS analysis of organic additives, quality control (QC) samples were injected every 10 samples to monitor system stability. Retention time drift was maintained within ± 0.1 min, and mass accuracy was kept within ±5 ppm. Internal standards were spiked into each sample to monitor extraction efficiency and instrument performance. The relative standard deviation of the internal standards remained below 15% throughout the analytical sequence.

For cell culture experiments, all reagents were tested for endotoxin contamination using the LAL assay. Mycoplasma testing was performed monthly using PCR‐based detection. Cell line authentication was conducted using short tandem repeat (STR) profiling before experiments. For viability assays, positive and negative controls were included on each plate, and edge wells were avoided to prevent edge effects. Z‐factors were calculated for each plate to ensure assay quality, with acceptance criteria of Z' > 0.5.

RNA sequencing quality control included RNA integrity number (RIN) assessment (minimum RIN > 8), library quality control using Bioanalyzer, and sequencing quality metrics (Q30> 80%, mapped reads > 80%). For differential expression analysis, batch effects were monitored using principal component analysis, and technical replicates showed Pearson correlation coefficients > 0.95. The sequencing depth was monitored to ensure >20 million uniquely mapped reads per sample.

For animal experiments, strict measures were implemented to minimize environmental contamination and ensure data integrity. Diets and water were supplied in glass or stainless‐steel containers to avoid plastic leaching. All gavage and necropsy procedures were conducted in clean rooms with minimal airflow disturbances, using metal or glass instruments exclusively. Personnel wore particle‐free protective clothing, including masks and nitrile gloves, and gloves were changed between handling individual animals to prevent cross‐contamination. During necropsy, tissue samples were collected on pre‐cleaned glass surfaces. Tissues were rinsed with filtered saline (0.22 µm) and stored in glass containers or pre‐cleaned metal cryovials to prevent the introduction of microplastics. Procedural blanks were included for each batch of tissue processing to monitor background contamination levels. For toxicological endpoint assessments (e.g., histopathology), positive and negative controls were included. All procedures complied with institutional animal care guidelines and were approved by the relevant ethics committee.

Statistical quality control included testing for normality, homoscedasticity, and outliers before analysis. Power calculations were performed to ensure adequate sample sizes for detecting biologically meaningful differences. For multiple comparisons, appropriate corrections were applied to control for false discovery rates. All statistical analyses were performed using validated methods and software versions, and two researchers independently verified the results.

All experimental procedures were documented in detail, including the lot numbers of reagents, instrument parameters, and any deviations from standard protocols. Raw data were backed up in triplicate, and all analysis scripts were version‐controlled. Regular external quality assessment was performed through participation in inter‐laboratory comparison studies where available.

### 4.15 Statistical Analyses

Statistical analyses were performed in R (version 4.4.1) with a predetermined significance level of α = 0.05. Sample sizes were determined by power analysis (β = 0.80) using preliminary data, yielding n = 3 for in vitro experiments (detecting 50% differences) and n≥5 for animal studies (detecting 30% differences). Normality was assessed using Shapiro‐Wilk tests. For normally distributed data, parametric tests (Student's *t*‐test and one‐way ANOVA) were used; for non‐normal data, non‐parametric tests (Mann–Whitney U and Kruskal–Wallis) were applied. Multiple comparisons were handled using the Benjamini–Hochberg false discovery rate (FDR) procedure, with Tukey's honestly significant difference (HSD) test following one‐way ANOVA when homogeneity of variance was met and the Games–Howell post hoc test when variances were unequal. Effect sizes were calculated using Cohen's d for t‐tests and eta‐squared (η^2^) for ANOVA. For particle size distributions, the Kolmogorov‐Smirnov tests were supplemented with effect‐size measures (D statistics). All data are presented as mean ± standard deviation (SD) unless otherwise specified. Error propagation for calculated values (e.g., oil film volume) was performed using the standard propagation‐of‐uncertainty equations.

## Author Contributions

C.J., R.X., and M.X. conceptualized the study and designed the research. R.X. and M.X. performed the experiments, visualized and analyzed the data, validated the findings, and drafted the original manuscript. G.Y., Q.C., Z.L., M.Y., W.G., D.L., Y.W., H.X., W.Z., Y.L., and C.P. contributed to experiments, data curation, validation, and manuscript review and editing. P.G., Q.L., T.Y., and X.F. contributed to methodology development, manuscript review, and editing. C.J. and M.X. supervised the project, secured funding, and reviewed and edited the manuscript.

## Funding

National Natural Science Foundation of China (NSFC) (82341109, 82173645, U21A20356, and 31371417), National Science and Technology Major Project of China (2025ZD0549000/2025ZD0549002), ZNSF (LR26C010001), and the Fundamental Research Funds for the Central Universities.

## Conflicts of Interest

The authors declare no conflicts of interest.

## Supporting information




**Supporting File 1**: advs74880‐sup‐0001‐SuppMat.docx.

**Supporting File 2**: advs74880‐sup‐0002‐TableS9.xls.

**Supporting File 3**: advs74880‐sup‐0003‐TableS10.xls.

**Supporting File 4**: advs74880‐sup‐0004‐TableS11.xls.

## Data Availability

The raw sequencing reads were submitted to ENA under project ID PRJEB84915. Raw microscopy images and source data supporting the findings of this study have been deposited in the Figshare repository (https://doi.org/10.6084/m9.figshare.31520173).
