# Supplementary material for: Oil‐Coated Nanoplastics Induce Rapid Membrane Disruption and Severe Intestinal Injury
Source: Adv Sci (Weinh). 2026 Mar 18;13(31):e20935. doi: 10.1002/advs.202520935 (PMC13587963; doi:10.1002/advs.202520935)
Supplement: Supplementary file 1 [file ADVS-13-e20935-s002.docx]

**Supplementary Information**

**Oil-coated nanoplastics induce rapid membrane disruption and severe intestinal injury**

Ruwen Xie^1^, Gulimire Yilihan^1^, Qiong Chen^1^, Dachuan Lin^1^, Zhen Liu^1^, Mengyi Yuan^1^, Yujia Wang^1^, Haoteng Xu^1^, Weishang Zhou^1^, Wanxin Gong^1^, Yueer Li^1^, Chen Peng^1^, Tong Yang^2^, Peng Gao^2^, Qing Liu^3,4^, Xin-Hua Feng^1,5^, Mu Xiao^1,5^*, Chao Jiang^1,6^*

^1^MOE Key Laboratory of Biosystems Homeostasis & Protection, and Zhejiang Key Laboratory of Molecular Cancer Biology, Life Sciences Institute, Zhejiang University, Hangzhou 310030, China

^2^Department of Environmental Health, Harvard T.H. Chan School of Public Health, Harvard University, Boston, MA 02115, USA

^3^Department of Biological Sciences, Clemson University, Clemson, SC 29634, USA

^4^Center for Human Genetics, Clemson University, Greenwood, SC 29646, USA

^5^Cancer Center, Zhejiang University, Hangzhou, Zhejiang 310058, China

^6^State Key Laboratory for Diagnosis and Treatment of Infectious Diseases, First Affiliated Hospital, Zhejiang University School of Medicine, Hangzhou, Zhejiang 310009, China

*Corresponding authors: Chao Jiang ([jiang_chao@zju.edu.cn](mailto:jiang_chao@zju.edu.cn)) and Mu Xiao (mu_xiao@zju.edu.cn)

# Supplementary figures

**Figure S1. Representative SEM images of PP- and PE-coated plastic containers’ inner walls after different treatments (A, C: microwave heating, D-F: transportation, G-H: leftover).** (a) PP-coated containers and (b) PE-coated containers’ inner walls in contact with DI water and soybean oil. Each experiment was repeated three times.

**Figure S2. Characterization of microplastics (MPs) and nanoplastics (NPs) released from different containers treated with DI water and soybean oil.** (a) PP and PE-coated containers were filled with 100 mL of soybean oil. (b) Optical microscope images (20x magnification) of plastic particles released from PP and PE-coated containers were captured directly in soybean oil under various experimental conditions. (c) SEM images depict PP MPs, PP NPs, PE MPs, and PE NPs released from different containers treated with DI water and soybean oil. (d) Raman spectroscopy profiles show the plastics released from PP and PE-coated containers in contact with soybean oil under various treatments. (e) Visualization of MPs released from PP containers and (f) PE-coated containers under different usage scenarios in contact with soybean oil and DI water. Representative images were generated from all experimental groups, comprising 28 merged images captured at 4x magnification using the montage function. The experiments were repeated independently three times.

**Figure S3. The abundance and size distribution of PP and PE MNPs in different treatment groups.** The abundance of (a) PP MPs and NPs and (b) PE MPs and NPs released from PP and PE-coated containers under experimental conditions in Groups D-H. (c) Size distribution of PP MPs and PP NPs and (d) PE MPs and PE NPs released from PP and PE-coated containers in Groups D-H. Sample sizes: MPs (n = 10 per group) and NPs (n = 9 per group). (e) PP and PE samples were prepared for Py-GC/MS analysis, along with the generated standard curves of PP and PE. The *p*-values for statistical differences were assessed using the two-sample Kolmogorov-Smirnov tests and **Student's t-test**s where applicable (**p* < 0.05; ***p* < 0.01; ****p* < 0.001). Data are expressed as the mean ± standard deviation.

**Figure S4. Chemical and physical alterations of MNPs released from plastic containers after oil-based heating.** (a) Abundance and (b) size distribution of MPs and NPs released from PP containers into different oils (n = 9 to 10 per group). (c) Clustered heatmap of all chemical features for PP, oil-PP MNPs, PE, and oil-PE MNPs samples, and abundance of all chemical features per sample (n = 3 per group). (d) consistently appearing chemical features between PP and oil-PP MNPs samples, as well as PE and oil-PE MNPs samples (n = 3 per group). (e) Heatmap of annotated chemicals categorized by class and superclass (n = 3 per group). The *p*-values for statistical differences were assessed using the one-way ANOVA test with post hoc Tukey tests. Different letters above each bar indicate *p* < 0.05.

**Figure S5. Comprehensive characterization of PP MPs and NPs released into various oils and aqueous media.** (a) SIM image of FL-oil-PP NPs (scale bar: 5 μm). (b) Size distribution of oil-PP NPs in different solvents detected by NTA. (c) Size distribution of various oil-PP NPs measured by DLS. (d) Z-average size and (e) PDI of different types of PP NPs dispersed in various solvents. (f) PDI and (g) size distribution of different oil-derived PP NPs. The *p*-values for statistical differences were assessed using the Kruskal-Wallis test where applicable. The data in S5d-S5f are presented as mean ± standard deviation, n = 3 per group.

**Figure S6. Dose-dependent KEGG pathway enrichment analysis after exposure of oil-PP NPs and water-PP NPs for different durations.** (a) Gene-function integration map tree showing differentially enriched pathways with significant (|ReporterScore| > 2, *p*.adj < 0.05) increases (orange) or decreases (green) in 20 hours of exposure to oil-PP NPs. (b) Bar plot displaying pathways with significant (|ReporterScore| > 2, *p*.adj < 0.05) increases (yellow) or decreases (blue) after 4 hours of exposure to water-PP NPs. (c) Bar plot displaying pathways with significant (|ReporterScore| > 2, p.adj < 0.05) increases (yellow) or decreases (blue) after 20 hours of exposure to water-PP NPs. (d) Venn diagram depicting up-regulated KEGG pathways (|ReporterScore| > 1.64, *p*.adj < 0.05) after 4 hours and 20 hours exposure with oil- and water-PP NPs. (e) Venn diagram of down-regulated KEGG pathways (ReporterScore  <-1.64, *p*.adj  < 0.05) after 4 hours and 20 hours of exposure to oil- and water-PP NPs. n = 3 per group.

**Figure S7. Significantly enriched KEGG pathways in response to oil-PP NPs exposure.** (a) Box charts of the up-regulated and down-regulated KEGG pathways after 4 hours of exposure to oil-PP NPs. (b) Box charts of the up-regulated and down-regulated KEGG pathways in response to 20 hours of exposure to oil-PP NPs. n = 3 per group.

**Figure S8. Significantly enriched KEGG pathways in response to water-PP NPs exposure.** (a) Box charts of up-regulated and down-regulated KEGG pathways after to 4 hours of exposure to water-PP NPs. (b) Box charts of up-regulated and down-regulated KEGG pathways after 20 hours of exposure to water-PP NPs. n = 3 per group.

**Figure S9.** **Up- and Down-regulated KEGG pathways in oil-PP NPs and water-PP NPs-treated groups.** Bubble plot showing (a) up- and (b) down-regulated KEGG pathways for oil-PP NPs vs. control and water-PP NPs vs. control in the 50 μg/mL, 4-hour treatment groups (ReporterScore < -2.5, *p* < 0.05). Bubble plot showing (c) up- and (d) down-regulated KEGG pathways for oil-PP NPs vs. control and water-PP NPs vs. control in the 10 μg/mL, 20-hour treatment groups (ReporterScore < -2.5, *p* < 0.05). n = 3 per group.

**Figure S10. Structural and physicochemical changes of oil-PP MNPs during simulated gastrointestinal digestion.** (a) Zeta potential of oil-PP MPs and NPs before and after exposure to simulated saliva, gastric juice, and intestinal juice. (b) Polydispersity index (PDI) of oil-PP MPs and NPs during digestion. (c) Size distribution of oil-PP NPs at different digestive stages. (d) ATR-FTIR spectra of oil-PP NPs across digestion stages. (e) TEM images of oil-PP MPs (top row) and oil-PP NPs (bottom row) before and after digestion. The data in S10a-S10b are presented as the mean ± standard deviation, n = 3 per group. Statistical differences were assessed using the Kruskal-Wallis tests.

**Figure S11. Oil-PP NP-induced changes in mouse body weight and intestinal immune transcriptome.** (a) Body weight changes in mice exposed to oil-PP NPs by gavage at doses of 10 mg/kg and 50 mg/kg compared with controls (n = 12 per group). (b) Principal component analysis (PCA) of intestinal transcriptome profiles from control and 50 mg/kg oil-PP NP-exposed mice. Distinct clustering of exposed and control groups indicates significant transcriptomic alterations (n = 6 per group). (c) Heatmaps of differentially expressed genes (DEGs) involved in tight junction integrity (left), T cell receptor signaling pathway (middle), and Nod-like receptor signaling pathway (right; n = 6 per group). (d) Immune-related gene expression trends categorized as adaptive, innate, or both, for the *in vitro* study (left) and *in vivo* study (right). Bars indicate the number of genes upregulated or downregulated in each category. (e) PCA of jejunal RNA-seq profiles from control, oil-PP NPs, and intervention groups (oil-PP NPs+MDP, +NaB, +β-glucan; n = 6 per group). (f) Schematic of the NOD2–RIPK2–TAB1–CHUK signaling axis targeted by MDP (left) and boxplots showing jejunal expression of *Nod2*, *Ripk2*, *Tab1*, and *Chuk* in oil-PP NP versus oil-PP NP+MDP mice (right; n = 6 per group). Statistical differences were assessed using the two-way ANOVA test and Wilcoxon tests where applicable (**p* < 0.05; ***p* < 0.01; ****p* < 0.001).

**Figure S12. Gene-based BMDL results and comparative analysis of heavy metal and MNPs intake across regions and exposure conditions.** (a, b) *In vitro* transcriptomic benchmark dose (BMD) and benchmark dose lower confidence limit (BMDL) for oil-PP NPs after 20 h exposure (n = 3 per group). (a) Scatter plot of 214 dose-responsive genes by KEGG category; (b) Boxplots for six selected genes showing TPM across concentrations. (c, d) *In vitro* transcriptomic BMD and BMDL for water-PP NPs after 20 h exposure (n = 3 per group). (c) Scatter plot of 596 dose-responsive genes; (d) Boxplots for six selected genes. (e, f) *In vivo* jejunal transcriptomic BMD and BMDL from mice gavaged with oil-PP NPs (0, 10, 50 mg/kg every three days for two weeks; n = 6 per group). (e) Scatter plot of 662 dose-responsive genes; (f) Boxplots for six selected genes. (g) Annual per-capita intake of PP and PE MNPs and co-released heavy metals (Cd, Cr, Ni, Pb) in adults and kids, modeled for three takeout-ordering frequencies (daily, weekly, monthly; n = 3 per group). (h) Annual PP MNP intake (g person⁻¹ year⁻¹) across 23 countries, with Shapiro–Wilk tests for normality (*p* = 0.027). (i) Comparison of PP MNPs intake between developed and developing countries (Wilcoxon test, *p* = 0.0001).

# Supplementary tables

**Table S1.** Py-GC/MS method performance and validation

| **Plastic** | **PP** | **PE** |
| --- | --- | --- |
| Linear range (mg/mL) | 0.02-1 | 0.02-1 |
| Recovery (%) | 95.63 | 123.13 |
| Calibration function | y=1909296.29⋅x−7353.92 | y=4708424.34⋅x−91953.81 |
| Linearity (R^2^) | 0.997 | 0.994 |
| RSD (%) | 5.43 | 10.38 |
| LOD (S/N=3) (mg/mL) | 0.061 | 0.094 |
| LOQ (S/N=10) (mg/mL) | 0.185 | 0.284 |

**Table S2.** Number of features and identified compounds in the samples

| **Polymer set** | **Total number of chemical features** | **Tentatively identified chemical features** | **Tentatively identified compounds** |
| --- | --- | --- | --- |
| PP | 296 | 12 | 12 |
| oil-PP MNPs | 963 | 48 | 34 |
| PE | 1682 | 93 | 70 |
| oil-PE MNPs | 2522 | 157 | 131 |
| Total | 3126 | 190 | 159 |

**Table S3.** Tentatively identified chemicals among the top ten features per sample.

| **CAS** | **Name** | **High abundance in the sample** | **Plastic related** | **Use** | **Toxicity** |
| --- | --- | --- | --- | --- | --- |
| 112-84-5 | Erucamide | PP, oil-PP MNPs, oil-PE MNPs | yes (A, B) | Colorant, filler, lubricant, food contact materials (B) | Irritant (C). Not classified as of potential concern (B) |
| 3886-90-6 | N,N-Dimethylstearamide | PP | yes (A, B) | Cosmetics (C) | Corrosive, irritant, health hazard (C) |
| 112-86-7 | cis-13-Docosenoic acid (Erucic Acid) | PP, oil-PP MNPs | yes (A, B) | Softener and conditioner, fillers, softener and conditioner, lubricating agent (C), plasticizer (B) | Irritant (C). RXR, ERa and ERb agonist, FXR, PPARg and ERR antagonist (D) |
| 11099-07-3 | 2,3-Dihydroxypropyl octadecanoate (Glyceryl Monostearate) | PP | yes (B, C) | Cosmetics, plasticizer (C) | Not classified as of potential concern (B) |
| - | Anticopalic acid (Copaiferic acid B) | PP | - | - | - |
| 56219-06-8 | Methyl myristoleate | PP | - | - | - |
| 479413-68-8 | 12-[(Cyclohexylcarbamoyl)amino]dodecanoic acid | PP | - | - | - |
| 667440-80-4 | 2-(2,3-Dimethylphenoxy)-2-methylpropanoic acid | PP | - | - | - |
| 23665-96-5 | Neogrifolin | PP | - | - | - |
| 4838-66-8 | 2-[2-(2-Quinolinyl)ethenyl]phenol | PP | - | - | - |
| 70356-09-1 | Avobenzone | oil-PP MNPs, oil-PE MNPs | yes (B) | Light stabilizer (B). Photosensitive chemicals, fragrance (C). UV stabilizer (D) | Irritant, health hazard (C). ERR ERa antagonist, PXR and CAR agonist (D) |
| [163520-33-0](https://commonchemistry.cas.org/detail?cas_rn=163520-33-0) | Isoxadifen-ethyl | oil-PP MNPs, oil-PE MNPs | - | Pesticides (C) | Irritant, environmental hazard (C). PXR agonist (D) |
| 68605-14-1 | Methyl octadecadienoate (Methyl linoleate) | oil-PP MNPs, PE | - | Cosmetics (C) | Not classified (C) |
| 54-28-4 | gamma-Tocopherol | oil-PP MNPs | yes (B) | Antioxidant (B). Food additives, fragrance (D) | Not classified (C). AR, ERa, and PPARg agonist, GR and ERa antagonist (D) |
| - | Oleoyl ethylamide (N-Ethyl-9Z-octadecenamide) | oil-PP MNPs | - | - | - |
| [3443-82-1](https://commonchemistry.cas.org/detail?cas_rn=3443-82-1) | 2-Linoleoylglycerol | oil-PP MNPs | - | - | - |
| 538-23-8 | Glycerol tricaprylate | oil-PP MNPs, oil-PE MNPs | yes (B) | Colorant, lubricant (B). Cosmetics (C) | Not classified (C) |
| 1643-20-5 | N,N-Dimethyldodecylamine N-oxide (Lauramine oxide) | oil-PP MNPs | yes (A, B) | Foaming, emulsifier, surface active agents (C) | Corrosive, irritant, environmental hazard (C) |
| [471-77-2](https://commonchemistry.cas.org/detail?cas_rn=471-77-2) | Neoabietic acid | PE | - | - | Irritant (C) |
| [3443-82-1](https://commonchemistry.cas.org/detail?cas_rn=3443-82-1) | 2-Linoleoylglycerol | PE | - | - | - |
| [17364-16-8](https://commonchemistry.cas.org/detail?cas_rn=17364-16-8) | 1-Palmitoyl-sn-glycero-3-phosphocholine | PE | - | Food additives and ingredients (C) | - |
| 24570-01-2 | 11beta-Prostaglandin E1 | PE | - | - | - |
| 25496-72-4 | Monoolein (Glyceryl monooleate) | PE | yes (B) | Lubricant additives, emulsifier, lubricating agents, surface active agents (C) | Not classified (C), HSE and PPARd agonist (D) |
| [506-21-8](https://commonchemistry.cas.org/detail?cas_rn=506-21-8) | Linoelaidic acid | PE | yes (A) | Lubricant (A).Cosmetics (C) | Not classified (C) |
| [1157-39-7](https://commonchemistry.cas.org/detail?cas_rn=1157-39-7) | 4',7-Dimethoxyisoflavone | PE | - | - | - |
| - | 13-Hydroxy-7-oxoabiet-8(14)-en-18-oic acid | PE | - | - | - |
| [68171-52-8](https://commonchemistry.cas.org/detail?cas_rn=68171-52-8) | Linoleoyl ethanolamide | PE | tentative | Surfactant (C). Softener and conditioner (D) | - |
| [3443-82-1](https://commonchemistry.cas.org/detail?cas_rn=3443-82-1) | 2-Linoleoylglycerol | oil-PE MNPs | - | - | - |
| [471-77-2](https://commonchemistry.cas.org/detail?cas_rn=471-77-2) | Neoabietic acid | oil-PE MNPs | - | - | Irritant (C) |
| [17364-16-8](https://commonchemistry.cas.org/detail?cas_rn=17364-16-8) | 1-Palmitoyl-sn-glycero-3-phosphocholine | oil-PE MNPs | - | Food additives and ingredients (C) | - |
| [60514-48-9](https://commonchemistry.cas.org/detail?cas_rn=60514-48-9) | 1,2-Dioctanoyl-sn-glycerol | oil-PE MNPs | - | - | - |
| 117-81-7 | DEHP | oil-PE MNPs | yes (B, C) | Binder, plasticizer (C) | Health hazard (C) |
| 22252-07-9 | 1-Linoleoyl-sn-glycero-3-phosphorylcholine | oil-PE MNPs | - | Food additives and ingredients (C) | - |

The usage and toxicity data were retrieved from multiple sources, including Sapozhnikova et al. (2021) ^1^, which focused on plastic chemicals migrating from food contact materials used in oven and microwave applications (A); Wiesinger et al. (2021)^2^, which detailed intentionally added plastic chemicals (B); as well as PubChem (C) and ToxCast (D).

**Table S4**. Oil film volume in a 200 μL suspension of oil-PP NPs at different concentrations.

| **Concentration (μg/mL)** | 2 | 5 | 10 | 20 | 30 | 50 | 70 | 100 | 200 | 500 | 1000 | 2000 |
| --- | --- | --- | --- | --- | --- | --- | --- | --- | --- | --- | --- | --- |
| **Oil film volume (μL)** | 0.005 | 0.013 | 0.027 | 0.054 | 0.081 | 0.135 | 0.190 | 0.271 | 0.542 | 1.353 | 2.708 | 5.415 |

Error propagation for the oil film volume per nanoparticle: 5.16×10^−12^ μL

**Table S5**. Adults’ and kids’ daily fat intake mass.

| **Type** | **Body weight (kg)** | **Daily calorie intake^1^ (kcal)** | **Daily fat intake^2^ (kcal)** | **Daily fat intake^3^ (g)** | **Fat intake per meal^4^ (g)** |
| --- | --- | --- | --- | --- | --- |
| Adult (18-59 years old) | 70 | 2500 | 750 | 84.8 | 33.9 |
| Kid (0-3 years old) | 12 | 1200 | 360 | 40.7 | 16.3 |

(1) Adult (18-59 years old) and kid (0-3 years old) daily calorie intake recommendations were provided by the Food and Agriculture Organization of the United Nations (FAO). (2) The World Health Organization (WHO) recommends reducing total fats to less than 30% of total energy intake. (3) Soybean oil provides 884 kilocalories per 100 grams; daily fat intake was calculated as soybean oil. (4) To calculate the daily fat intake in grams from soybean oil, lunch consumption was considered, accounting for 40% of the total daily food intake, based on the dietary distribution of 30% for breakfast, 40% for lunch, and 30% for dinner.

**Table S6.** Estimated annual takeout frequency, oil-PP MNPs Intake, and five-year intestinal accumulation for 1 µm and 200 nm particles across 23 regions

| **Country** | **Continent** | **Development Status** | **Average take-out food ordering frequency (per week)** | **Mass of intake oil-PP MNPs (g/year)** | $\boldsymbol{R}_{\boldsymbol{in}}$ **(1 μm)**  **(µg·g⁻¹·year⁻¹)** | $\boldsymbol{R}_{\boldsymbol{in}}$ **(200 nm)**  **(µg·g⁻¹·year⁻¹)** | $\boldsymbol{C}_{\boldsymbol{5}}$ **(1 μm)**  **(µg·g⁻¹)** | $\boldsymbol{C}_{\boldsymbol{5}}$ **(200 nm)**  **(µg·g⁻¹)** |
| --- | --- | --- | --- | --- | --- | --- | --- | --- |
| China | Asia | Developing | 4.459^a^ | 3.354 | 2.096 | 104.813 | 8.858 | 442.891 |
| South Korea | Asia | Developed | 0.963^a^ | 0.725 | 0.453 | 22.656 | 1.915 | 95.735 |
| Thailand | Asia | Developing | 3.407^a^ | 2.563 | 1.602 | 80.094 | 6.769 | 338.441 |
| Singapore | Asia | Developed | 1.720^a^ | 1.294 | 0.809 | 40.438 | 3.417 | 170.871 |
| Australia | Oceania | Developed | 0.909^a^ | 0.684 | 0.428 | 21.375 | 1.806 | 90.321 |
| USA | North America | Developed | 1.527^b^ | 1.149 | 0.718 | 35.906 | 3.034 | 151.724 |
| UK | Europe | Developed | 0.568^b^ | 0.427 | 0.267 | 13.344 | 1.128 | 56.385 |
| Egypt | Africa | Developing | 2.238^b^ | 1.683 | 1.052 | 52.594 | 4.445 | 222.238 |
| Mexico | North America | Developing | 1.965^b^ | 1.478 | 0.924 | 46.188 | 3.903 | 195.168 |
| Italy | Europe | Developed | 1.048^b^ | 0.788 | 0.493 | 24.625 | 2.081 | 104.054 |
| Canada | North America | Developed | 1.214^b^ | 0.913 | 0.571 | 28.531 | 2.411 | 120.560 |
| Saudi Arabia | Asia | Developing | 2.887^b^ | 2.172 | 1.358 | 67.875 | 5.736 | 286.810 |
| South Africa | Africa | Developing | 2.695^b^ | 2.028 | 1.268 | 63.375 | 5.356 | 267.795 |
| Germany | Europe | Developed | 0.848^b^ | 0.638 | 0.399 | 19.938 | 1.685 | 84.247 |
| Spain | Europe | Developed | 0.976^b^ | 0.734 | 0.459 | 22.938 | 1.938 | 96.924 |
| Indonesia | Asia | Developing | 2.591^b^ | 1.949 | 1.218 | 60.906 | 5.147 | 257.363 |
| UAE | Asia | Developed | 2.620^b^ | 1.971 | 1.232 | 61.594 | 5.205 | 260.268 |
| Russia | Europe | Developing | 1.593^c^ | 1.198 | 0.749 | 37.438 | 3.164 | 158.194 |
| Brazil | South America | Developing | 1.145^d^ | 0.861 | 0.538 | 26.906 | 2.274 | 113.694 |
| Vietnam | Asia | Developing | 3.006^b^ | 2.278 | 1.424 | 71.188 | 6.016 | 300.807 |
| India | Asia | Developing | 2.105^b^ | 1.595 | 0.997 | 49.844 | 4.212 | 210.618 |
| France | Europe | Developed | 0.696^b^ | 0.528 | 0.330 | 16.500 | 1.394 | 69.722 |
| Japan | Asia | Developed | 0.719^b^ | 0.545 | 0.341 | 17.031 | 1.439 | 71.966 |

The average frequency of take-out food ordering in different regions was calculated based on the data collected from (a) Statista, (b) YouGov, (c) Yandex, and (d) Opinion box.

**Table S7**. Calculated five-year small-intestine threshold concentrations based on mouse gavage BMDL doses.

| $\boldsymbol{BMDL}_{\boldsymbol{gavage}}$**(mg/kg)** | **Human equivalent dose (HED**, **mg/kg·day)** | **Estimated daily intake (**$\boldsymbol{D}^{\boldsymbol{*}}$**,μg/day)** | **Five-year small-intestine accumulation threshold (**$\boldsymbol{C}_{\boldsymbol{5}}^{\boldsymbol{th}}\boldsymbol{200 nm}$**, μg/g)** |
| --- | --- | --- | --- |
| 33.86 | 0.92 | 54908.11 | 3131.48 |
| 23.34 | 0.63 | 37848.65 | 2158.56 |
| 19.91 | 0.54 | 32286.49 | 1841.34 |
| 18.36 | 0.50 | 29772.97 | 1697.99 |
| 15.83 | 0.43 | 25670.27 | 1464.01 |
| 33.33 | 0.9033 | 54195.12 | 2612.22 |
| 20.36 | 0.5518 | 33105.69 | 1595.73 |
| 5.93 | 0.1607 | 9642.28 | 464.72 |
| 5.89 | 0.1596 | 9577.24 | 461.54 |
| 5.58 | 0.1512 | 9073.17 | 437.25 |
| 4.8 | 0.1301 | 7804.88 | 376.23 |

$\boldsymbol{BMDL}_{\boldsymbol{gavage}}$: Benchmark dose lower limit from mouse gavage experiments (mg of MNPs per kg body weight). **HED (mg/kg·day)**: Human equivalent dose per day, converted from mouse gavage BMDL using allometric scaling. $\boldsymbol{D}^{\boldsymbol{*}}$ **(μg/day)**: Estimated daily intake for a 60-kg adult, derived from the HED. $\boldsymbol{C}_{\boldsymbol{5}}^{\boldsymbol{th}}\boldsymbol{200 nm}$ **(μg/g)**: Modeled five-year small-intestine accumulation threshold concentration for 200 nm particles (µg MNPs per g intestinal tissue).

**Table S8.** Presence of MNPs in human tissues or fluids.

| **Human biological sample** | **Physical condition** | **Main type** | **Abundance (range or average)** | **Reference** |
| --- | --- | --- | --- | --- |
| Blood | Healthy | PET, PS, and PE | 1.6 μg/mL | ^3^ |
| Testis | Patients | PE, ABS, N66, PS, PET, and PVC | 15.34 ± 23.31 μg/mL, 328.4 μg/g: 18.12 (PP), 115.75 (PE) | ^4,5^ |
| Bone marrow | Patients | PE, PS, PVC, PA66, and PP | 51.3 μg/g: 1.75 µg/g (PP), 30.02 µg/g (PE) | ^6^ |
| Cumulus granulosa cell | Female patients of infertile couples | PET | 313.1 ± 246.7 µg/g | ^7^ |
| Follicular fluid |  | / | 12.9 ± 15.5 μg/g |  |
| Placenta | Healthy | PE, PVC, and PA | 126.8 ± 147.5 µg/g: 1.35 µg/g (PP), 68.8 µg/g (PE) | ^8^ |
| Balf | Patients | PP, and PE | 7.1μg/g (PE); 1.1 μg/g (PP) | ^9^ |
| Gallstone | Patients | PP, PE, and PET | 0.09 µg/g (PP), 3.56 µg/g (PE) | ^10^ |
| Feces (adult) | Healthy | PET, PC, PE, and PP | 2.2–16.0 μg/g (PET); 0.037–0.62 μg/g (PC), 3.48−9.2 μg/g (PE), 3.13−10.19 µg/g (PP) | ^11–13^ |
| Feces (infant) | Healthy | PET and PC | 5.70–82.0 μg/g (PET); 0.049–2.10 μg/g (PC) |  |
| Carotid artery plaque | Patients | PE (58.4%), PVC (12.1%) | 21.7 ± 24.5 (PE); 5.2 ± 2.4 μg/mg (PVC) | ^14^ |
| Atherosclerotic plaque | Patients | PET, PA, PVC, and PE | 118.66 ± 53.87ug/g | ^15^ |
| Thrombi | Patients | PE, PA66, and PVC | 14.9–221.8 μg/g | ^16^ |
| Cervix | Patients | PE, PP, and PE-co-PP | 1.67 ± 0.94 (Cancer tissue); | ^17^ |
|  |  | PE, PP, and PE-co-PP | 0.87 ± 0.72 µg/g (Paracancerous tissue) |  |
| Liver | Healthy | PE, PP, PMMA, and PC | 141.9 µg/g (2016); 465.3 µg/g (2024) | ^18^ |
| Kidney | Healthy | PE, PP, and SBR | 538.1 µg/g (2016); 666.3 µg/g (2024) |  |
| Brain | Healthy | PE, PP, and PVC | 3420 µg/g (2016); 4763 µg/g (2024) |  |

PE: polyethylene; PET: polyethylene terephthalate; PVC: polyvinyl chloride; PP: polypropylene; PS: polystyrene; PA: polyamide; PC: polycarbonate; PMMA: polymethyl methacrylate; ABS: Acrylonitrile Butadiene Styrene; N66: Nylon 66; SBR: styrene-butadiene rubber.

# Reference

1.	Sapozhnikova, Y., Nuñez, A. & Johnston, J. Screening of chemicals migrating from plastic food contact materials for oven and microwave applications by liquid and gas chromatography - Orbitrap mass spectrometry. *Journal of Chromatography A* **1651**, 462261 (2021).

2.	Wiesinger, H., Wang, Z. & Hellweg, S. Deep Dive into Plastic Monomers, Additives, and Processing Aids. *Environ. Sci. Technol.* **55**, 9339–9351 (2021).

3.	Leslie, H. A. *et al.* Discovery and quantification of plastic particle pollution in human blood. *Environment International* **163**, 107199 (2022).

4.	Zhao, Q. *et al.* Detection and characterization of microplastics in the human testis and semen. *Science of The Total Environment* **877**, 162713 (2023).

5.	Hu, C. J. *et al.* Microplastic presence in dog and human testis and its potential association with sperm count and weights of testis and epididymis. *Toxicological Sciences* **200**, 235–240 (2024).

6.	Guo, X. *et al.* Discovery and analysis of microplastics in human bone marrow. *Journal of Hazardous Materials* **477**, 135266 (2024).

7.	Weina, L. *et al.* P-762 Discovery and quantification of microplastics in human cumulus granulosa cells. *Human Reproduction* **39**, deae108.1081 (2024).

8.	Garcia, M. A. *et al.* Quantitation and identification of microplastics accumulation in human placental specimens using pyrolysis gas chromatography mass spectrometry. *Toxicological Sciences* **199**, 81–88 (2024).

9.	Zhong, C. *et al.* Nanoplastics in the Human Respiratory System. *Am J Respir Crit Care Med* **210**, 1059–1061 (2024).

10.	Zhang, D. *et al.* Microplastics are detected in human gallstones and have the ability to form large cholesterol-microplastic heteroaggregates. *Journal of Hazardous Materials* **467**, 133631 (2024).

11.	Luqman, A. *et al.* Microplastic Contamination in Human Stools, Foods, and Drinking Water Associated with Indonesian Coastal Population. *Environments* **8**, 138 (2021).

12.	Wibowo, A. T. *et al.* Microplastic Contamination in the Human Gastrointestinal Tract and Daily Consumables Associated with an Indonesian Farming Community. *Sustainability* **13**, 12840 (2021).

13.	Zhang, J., Wang, L., Trasande, L. & Kannan, K. Occurrence of Polyethylene Terephthalate and Polycarbonate Microplastics in Infant and Adult Feces. *Environ. Sci. Technol. Lett.* **8**, 989–994 (2021).

14.	Marfella, R. *et al.* Microplastics and Nanoplastics in Atheromas and Cardiovascular Events. *N Engl J Med* **390**, 900–910 (2024).

15.	Liu, S. *et al.* Microplastics in three types of human arteries detected by pyrolysis-gas chromatography/mass spectrometry (Py-GC/MS). *Journal of Hazardous Materials* **469**, 133855 (2024).

16.	Wang, T. *et al.* Multimodal detection and analysis of microplastics in human thrombi from multiple anatomically distinct sites. *eBioMedicine* **103**, 105118 (2024).

17.	Xu, H. *et al.* Detection and analysis of microplastics in tissues and blood of human cervical cancer patients. *Environmental Research* **259**, 119498 (2024).

18.	Nihart, A. J. *et al.* Bioaccumulation of microplastics in decedent human brains. *Nat Med* 1–6 (2025) doi:10.1038/s41591-024-03453-1.
